# Time-dependent solution of the NIMFA equations around the epidemic threshold

**DOI:** 10.1007/s00285-020-01542-6

**Published:** 2020-09-22

**Authors:** Bastian Prasse, Piet Van Mieghem

**Affiliations:** Faculty of Electrical Engineering, Mathematics and Computer Science, P.O Box 5031, 2600 GA Delft, The Netherlands

**Keywords:** NIMFA differential equations, SIS process, Epidemic models, Viral state dynamics, 92D30, 92D25, 34A34

## Abstract

The majority of epidemic models are described by non-linear differential equations which do not have a closed-form solution. Due to the absence of a closed-form solution, the understanding of the precise dynamics of a virus is rather limited. We solve the differential equations of the *N*-intertwined mean-field approximation of the susceptible-infected-susceptible epidemic process with heterogeneous spreading parameters around the epidemic threshold for an arbitrary contact network, provided that the initial viral state vector is small or parallel to the steady-state vector. Numerical simulations demonstrate that the solution around the epidemic threshold is accurate, also above the epidemic threshold and for general initial viral states that are below the steady-state.

## Introduction

Epidemiology originates from the study of infectious diseases such as gonorrhoea, cholera and the flu (Bailey 1975; Anderson and May 1992). Human beings do not only transmit infectious diseases from one individual to another, but also opinions, on-line social media content and innovations. Furthermore, man-made structures exhibit epidemic phenomena, such as the propagation of failures in power networks or the spread of a malicious computer virus. Modern epidemiology has evolved into the study of general spreading processes (Pastor-Satorras et al. 2015; Nowzari et al. 2016). Two properties are essential to a broad class of epidemic models. First, individuals are either infected with the disease (respectively, possess the information, opinion, etc.) or healthy. Second, individuals can infect one another only if they are in contact (e.g., by a friendship). In this work, we consider an epidemic model which describes the spread of a virus between groups of individuals.

We consider a *contact network* of *N* nodes, and every node $i=1,\ldots , N$ corresponds to a group^1^ of individuals. If the members of two groups *i*, *j* are in contact, then group *i* and group *j* can infect one another with the virus. We denote the symmetric $N \times N$ adjacency matrix by *A* and its elements by $a_{ij}$. If there is a link between node *i* and node *j*, then $a_{ij}=1$, and $a_{ij}=0$ otherwise. Hence, the virus directly spreads between two nodes *i* and *j* only if $a_{ij}=1$. We stress that in most applications it holds that $a_{ii}\ne 0$, since infected individuals in group *i* usually do infect susceptible individuals in the same group *i*. At any time $t\ge 0$, we denote the viral state of node *i* by $v_i(t)$. The viral state $v_i(t)$ is in the interval [0, 1] and is interpreted as the fraction of infected individuals of group *i*. *N*-intertwined mean-field approximation (NIMFA) with *heterogeneous* spreading parameters (Lajmanovich and Yorke 1976; Van Mieghem and Omic 2014) assumes that the curing rates $\delta _i$ and infection rates $\beta _{ij}$ depend on the nodes *i* and *j*.

### Definition 1

(*Heterogeneous NIMFA*) At any time $t\ge 0$, the NIMFA governing equation is

1 $$\begin{aligned} \frac{d v_i (t)}{d t }&= - \delta _i v_i(t) + \left( 1 - v_i(t)\right) \sum ^N_{j = 1} {\tilde{\beta }}_{i j} a_{ij} v_j(t) \end{aligned}$$

for every group $i=1,\ldots , N$, where $\delta _i >0$ is the curing rate of node *i*, and ${\tilde{\beta }}_{i j} > 0$ is the infection rate from node *j* to node *i*.

For a vector $x\in {\mathbb {R}}^N$, we denote the diagonal matrix with *x* on its diagonal by ${\text {diag}}(x)$. We denote the $N\times N$ curing rate matrix $S = {\text {diag}}(\delta _1,\ldots , \delta _N)$. Then, the matrix form of (1) is a vector differential equation

2 $$\begin{aligned} \frac{d v (t)}{d t }&= - S v(t) + {\mathrm{diag}}\left( u - v(t)\right) B v(t), \end{aligned}$$

where $v(t) = (v_1(t),\ldots , v_N(t))^T$ is the viral state vector at time *t*, the $N\times N$ infection rate matrix *B* is composed of the elements $\beta _{ij} = {\tilde{\beta }}_{ij} a_{ij}$, and *u* is the $N\times 1$ all-one vector. In this work, we assume that the matrix *B* is symmetric.

### Definition 2

(*Steady-State Vector*) The $N \times 1$ *steady-state vector* $v_\infty$ is the non-zero equilibrium of NIMFA, which satisfies

3 $$\begin{aligned} \left( B - S \right) v_\infty = {\mathrm{diag}}\left( v_\infty \right) B v_\infty . \end{aligned}$$

In its simplest form, NIMFA (Van Mieghem et al. 2009) assumes the same infection rate $\beta$ and curing rate $\delta$ for all nodes. More precisely, for *homogeneous NIMFA* the governing equations (2) reduce to

4 $$\begin{aligned} \frac{d v (t)}{d t }&= - \delta v(t) + \beta {\mathrm{diag}}\left( u - v(t)\right) A v(t). \end{aligned}$$

For the vast majority of epidemiological, demographical, and ecological models, the *basic reproduction number* $R_0$ is an essential quantity (Hethcote 2000; Heesterbeek 2002). The basic reproduction number $R_0$ is defined (Diekmann et al. 1990) as “The expected number of secondary cases produced, in a completely susceptible population, by a typical infective individual during its entire period of infectiousness”. Originally, the basic reproduction number $R_0$ was introduced for epidemiological models with only $N=1$ group of individuals. Van den Driessche and Watmough (2002) proposed a definition of the basic reproduction number $R_0$ to epidemic models with $N>1$ groups. For NIMFA (1), the basic reproduction number $R_0$ follows (Van den Driessche and Watmough 2002) as $R_0 = \rho (S^{-1}B)$, where $\rho (M)$ denotes the spectral radius of a square matrix *M*. For the stochastic Susceptible-Infected-Removed (SIR) epidemic process on data-driven contact networks, Liu et al. (2018) argue that the basic reproduction number $R_0$ is inadequate to characterise the behaviour of the viral dynamics, since the number of secondary cases produced by an infectious individual varies greatly with time *t*. In contrast to the stochastic SIR process, for the deterministic NIMFA equations (1), the basic reproduction number $R_0 = \rho (S^{-1}B)$ is of crucial importance for the viral state dynamics. Lajmanovich and Yorke (1976) showed that there is a phase transition at the *epidemic threshold criterion* $R_0 = 1$: If $R_0 \le 1$, then the only equilibrium of NIMFA (1) is the origin, which is globally asymptotically stable. Else, if $R_0 > 1$, then there is a second equilibrium, the steady-state $v_\infty$, whose components are positive, and the steady-state $v_\infty$ is globally asymptotically stable for every initial viral state $v(0) \ne 0$. For real-world epidemics, the regime around epidemic threshold criterion $R_0 = 1$ is of particular interest. In practice, the basic reproduction number $R_0$ cannot be arbitrarily great, since natural immunities and vaccinations lead to significant curing rates $\delta _i$ and the frequency and intensity of human contacts constrain the infection rates $\beta _{ij}$. Beyond the spread of infectious diseases, many real-world systems seem to operate in the critical regime around a phase transition (Kitzbichler et al. 2009; Nykter et al. 2008).

The basic reproduction number $R_0$ only provides a coarse description of the dynamics of NIMFA (1). Recently (Prasse and Van Mieghem 2019), we analysed the viral state dynamics for the *discrete-time* version of NIMFA (1), provided that the initial viral state *v*(0) is small (see also Assumption 2 in Sect. 3). Three results of Prasse and Van Mieghem (2019) are worth mentioning, since we believe that they could also apply to NIMFA (1) in continuous time. First, the steady-state $v_\infty$ is exponentially stable. Second, the viral state is (almost always) monotonically increasing. Third, the viral state *v*(*t*) is bounded by linear time-invariant systems at any time *t*. In this work, we go a step further in analysing the dynamics of the viral state *v*(*t*), and we focus on the region around the threshold $R_0 =1$. More precisely, we find the closed-form expression of the viral state $v_i(t)$ *for every node* *i* *at every time* *t* when $R_0 \downarrow 1$, given that the initial state *v*(0) is small or parallel^2^ to the steady-state vector $v_\infty$.

We introduce the assumptions in Sect. 3. Section 4 gives an explicit expression for the steady-state vector $v_\infty$ when $R_0 \downarrow 1$. In Sect. 5, we derive the closed-form expression for the viral state vector *v*(*t*) at any time $t\ge 0$. The closed-form solution for $R_0 \downarrow 1$ gives an accurate approximation also for $R_0 >1$ as demonstrated by numerical evaluations in Sect. 6.

## Related work

Lajmanovich and Yorke (1976) originally proposed the differential equations (1) to model the spread of gonorrhoea and proved the existence and global asymptotic stability of the steady-state $v_\infty$ for strongly connected directed graphs. In Lajmanovich and Yorke (1976), Fall et al. (2007), Wan et al. (2008), Rami et al. (2013), Prasse and Van Mieghem (2018) and Paré et al. (2018), the differential equations (1) are considered as the *exact* description of the virus spread between groups of individuals. Van Mieghem et al. (2009) derived the differential equations (1) as an *approximation* of the Markovian Susceptible-Infected-Susceptible (SIS) epidemic process (Pastor-Satorras et al. 2015; Nowzari et al. 2016), which lead to the acronym “NIMFA” for “*N*-Intertwined Mean-Field Approximation” (Van Mieghem 2011; Van Mieghem and Omic 2014; Devriendt and Van Mieghem 2017). The approximation of the SIS epidemic process by NIMFA is least accurate around the epidemic threshold (Van Mieghem et al. 2009; Van Mieghem and van de Bovenkamp 2015). Thus, the solution of NIMFA when $R_0\downarrow 1$, which is derived in this work, might be inaccurate for the description of the probabilistic SIS process.

Fall et al. (2007) analysed the generalisation of the differential equations (1) of Lajmanovich and Yorke (1976) to a non-diagonal curing rate matrix *S*. Khanafer et al. (2016) showed that the steady-state $v_\infty$ is globally asymptotically stable, also for weakly connected directed graphs. Furthermore, NIMFA (1) has been generalised to time-varying parameters. Paré et al. (2017) consider that the infection rates^3^$\beta _{ij}(t)$ depend continuously on time *t*. Rami et al. (2013) consider a switched model in which both the infection rates $\beta _{ij}(t)$ and the curing rates $\delta _i(t)$ change with time *t*. NIMFA (1) in discrete time has been analysed in Ahn and Hassibi (2013), Paré et al. (2018), Prasse and Van Mieghem (2019) and Liu et al. (2020).

In Van Mieghem (2014b), NIMFA (4) was solved for a special case: If the adjacency matrix *A* corresponds to a regular graph and the initial state $v_i(0)$ is the same^4^ for every node *i*, then NIMFA with time-varying, homogeneous spreading parameters $\beta (t), \delta (t)$ has a closed-form solution. In this work, we focus on time-invariant but heterogeneous spreading parameters $\delta _i, \beta _{ij}$. We solve NIMFA (1) for *arbitrary* graphs around the threshold criterion $R_0 = 1$ and for an initial viral state *v*(0) that is small or parallel to the steady-state vector $v_\infty$.

## Notations and assumptions

The basic reproduction number $R_0= \rho (S^{-1}B)$ is determined by the infection rate matrix *B* and the curing rate matrix *S*. Thus, the notation $R_0 \downarrow 1$ is imprecise, since there are infinitely many matrices *B*, *S* such that the basic reproduction number $R_0$ equals 1. To be more precise, we consider a sequence $\left\{ \left( B^{(n)}, S^{(n)}\right) \right\} _{n \in {\mathbb {N}}}$ of infection rate matrices $B^{(n)}$ and curing rate matrices $S^{(n)}$ that converges^5^ to a limit $(B^*, S^*)$, such that $\rho \left( \left( S^* \right) ^{-1}B^*\right) =1$ and

$$\begin{aligned} \rho \left( \left( S^{(n)} \right) ^{-1}B^{(n)}\right) > 1 \quad \forall n \in {\mathbb {N}}. \end{aligned}$$

For the ease of exposition, we drop the index *n* and replace $B^{(n)}$ and $S^{(n)}$ by the notation *B* and *S*, respectively. In particular, we emphasise that the assumptions below apply to every element $\left( B^{(n)}, S^{(n)}\right)$ of the sequence. In Sects. 4 to 6, we formally abbreviated the limit process $\left( B^{(n)}, S^{(n)}\right) \rightarrow \left( B^*, S^*\right)$ by the notation $R_0\downarrow 1$. For the proofs in the appendices, we use the lengthier but clearer notation $\left( B, S\right) \rightarrow \left( B^*, S^*\right)$. Furthermore, we use the superscript notation $\varXi ^*$ to denote the limit of any variable $\varXi$ that depends on the infection rate matrix *B* and the curing rate matrix *S*. For instance, $\delta ^*_i$ denotes the limit of the curing rate $\delta _i$ of node *i* when $\left( B, S\right) \rightarrow \left( B^*, S^*\right)$. The Landau-notation $f(R_0) = {\mathcal {O}}(g(R_0))$ as $R_0 \downarrow 1$ denotes that $|f(R_0)| \le \sigma |g(R_0)|$ for some constant $\sigma$ as $R_0 \downarrow 1$. For instance, it holds that $(R_0-1)^2 = {\mathcal {O}}( R_0-1 )$ as $R_0 \downarrow 1$.

In the remainder of this work, we rely on three assumptions, which we state for clarity in this section.

### Assumption 1

For every basic reproduction number $R_0>1$, the curing rates are positive and the infection rates are non-negative, i.e., $\delta _i >0$ and $\beta _{ij} \ge 0$ for all nodes *i*, *j*. Furthermore, in the limit $R_0\downarrow 1$, it holds that $\delta _i \not \rightarrow 0$ and $\delta _i \not \rightarrow \infty$ for all nodes *i*.

We consider Assumption 1 a rather technical assumption, since only non-negative rates $\delta _i$ and $\beta _{ij}$ have a physical meaning. Furthermore, if the curing rates $\delta _i$ were zero, then the differential equations (1) would describe a Susceptible-Infected (SI) epidemic process. In this work, we focus on the SIS epidemic process, for which it holds that $\delta _i>0$.

### Assumption 2

For every basic reproduction number $R_0>1$, it holds that $v_i(0) \ge 0$ and $v_i(0) \le v_{\infty , i}$ for every node $i=1,\ldots , N$. Furthermore, it holds that $v_i(0)>0$ for at least one node *i*.

For the description of most real-world epidemics, Assumption 2 is reasonable for two reasons. First, the total number of infected individuals often is small in the beginning of an epidemic outbreak. (Sometimes, there is even a single patient zero.) Second, a group *i* often contains many individuals. For instance, the viral state $v_i(t)$ could describe the prevalence of virus in municipality *i*. Thus, even if there is a considerable total number of infected individuals in group *i*, the initial *fraction* $v_i(0)$ would be small.

### Assumption 3

For every basic reproduction number $R_0>1$, the infection rate matrix *B* is symmetric and irreducible. Furthermore, in the limit $R_0\downarrow 1$, the infection rate matrix *B* converges to a symmetric and irreducible matrix.

Assumption 3 holds if and only if the infection rate matrix *B* (and its limit) corresponds to a connected undirected graph (Van Mieghem 2014a).

## The steady-state around the epidemic threshold

We define the $N\times N$ *effective infection rate matrix* *W* as

5 $$\begin{aligned} W = S^{-1} B. \end{aligned}$$

In this section, we state an essential property that we apply to solve the NIMFA equations (1) when the basic reproduction number $R_0$ is close to 1: *The steady-state vector* $v_\infty$ *converges to a scaled version of the principal eigenvector* $x_1$ *of the effective infection rate matrix* *W* *when* $R_0 \downarrow 1$.

Under Assumptions 1 and 3, the effective infection rate matrix *W* is non-negative and irreducible. Hence, the Perron–Frobenius Theorem (Van Mieghem 2014a) implies that the matrix *W* has a unique eigenvalue $\lambda _1$ which equals the spectral radius $\rho (W)$. As we show in the beginning of Appendix B, the eigenvalues of the effective infection rate matrix *W* are real and satisfy $\lambda _1 = \rho (W) > \lambda _2\ge \cdots \ge \lambda _N$. In particular, under Assumptions 1 and 3, the largest eigenvalue $\lambda _1$, the spectral radius $\rho (W)$ and the basic reproduction number $R_0$ are the same quantity, i.e., $R_0 = \rho (W) = \lambda _1$.

In Van Mieghem (2012, Lemma 4) it was shown that, for homogeneous NIMFA (4), the steady-state vector $v_\infty$ converges to a scaled version of the principal eigenvector of the adjacency matrix *A* when $R_0 \downarrow 1$. We generalise the results of Van Mieghem (2012) to heterogeneous NIMFA (1):

### Theorem 1

Under Assumptions 1 and 3, the steady-state vector $v_\infty$ obeys

6 $$\begin{aligned} v_\infty = \gamma x_1 + \eta , \end{aligned}$$

where the scalar $\gamma$ equals

7 $$\begin{aligned} \gamma = \left( R_0 - 1 \right) \frac{\sum ^N_{l=1} \delta _l \left( x_1\right) ^2_l}{\sum ^N_{l=1} \delta _l \left( x_1\right) ^3_l}, \end{aligned}$$

and the $N\times 1$ vector $\eta$ satisfies $\Vert \eta \Vert _2 \le {\mathcal {O}}\left( \left( R_0-1\right) ^2\right)$ when the basic reproduction number $R_0$ approaches 1 from above.

### Proof

Appendix B. $$\square $$

## The viral state dynamics around the epidemic threshold

In Sect. 5.1, we give an intuitive motivation of our solution approach for the NIMFA equations (1) when $R_0 \downarrow 1$. In Sect. 5.2, we state our main result.

### Motivation of the solution approach

For simplicity, this subsection is confined to the homogeneous NIMFA equations (4). In numerical simulations (Prasse and Van Mieghem 2018), we observed that the $N \times N$ viral state matrix $V=(v(t_1),\ldots , v(t_N))$, for arbitrary observation times $t_1<\cdots < t_N$, is severely ill-conditioned. Thus, the viral state *v*(*t*) at any time $t\ge 0$ approximately equals the linear combination of $m<<N$ orthogonal vectors $y_1,\ldots , y_m$, and we can write $v(t) \approx c_1(t) y_1 + \cdots + c_m(t) y_m$, see also Prasse and Van Mieghem (2020). Here, the functions $c_1(t),\ldots , c_m(t)$ are scalar. We consider the most extreme case by representing the viral state *v*(*t*) by a scaled version of only $m=1$ vector $y_1$, which corresponds to $v(t)\approx c(t) y_1$ for a scalar function *c*(*t*). The viral state *v*(*t*) converges to the steady-state vector $v_\infty$ as $t\rightarrow \infty$. Hence, a natural choice for the vector $y_1$ is $y_1 = v_\infty$, which implies that $c(t)\rightarrow 1$ as $t\rightarrow \infty$. If $R_0 \approx 1$ and $v(0)\approx 0$, then the approximation $v(t) \approx c(t) v_\infty$ is accurate *at all times* $t\ge 0$ due to two intuitive reasons.

1. If $v(t)\approx 0$ when $t \approx 0$, then NIMFA (4) is approximated by the linearisation around zero. Hence, it holds that
  8 $$\begin{aligned} \frac{d v (t)}{d t } \approx \left( \beta A - \delta I \right) v(t) \end{aligned}$$
  when $t\approx 0$. The state *v*(*t*) of the linear system (8) converges rapidly to a scaled version of the principal eigenvector $x_1$ of the matrix $\left( \beta A - \delta I \right)$. Furthermore, Theorem 1 states that $v_\infty \approx \gamma x_1$ when $R_0 \approx 1$. Thus, the viral state *v*(*t*) rapidly converges to a scaled version of the steady-state $v_\infty$:
2. Suppose that the viral state *v*(*t*) approximately equals to a scaled version of the steady-state vector $v_\infty$. (In other words, the viral state *v*(*t*) is “almost parallel” to the vector $v_\infty$.) Then, it holds that
  9 $$\begin{aligned} v(t) \approx c(t) v_\infty \end{aligned}$$
  for some scalar *c*(*t*). We insert (9) into the NIMFA equations (4), which yields that
  10 $$\begin{aligned} \frac{d c(t)}{d t } v_\infty&\approx c(t) \left( \beta A - \delta I \right) v_\infty - \beta c^2(t) {\text {diag}}(v_\infty ) A v_\infty . \end{aligned}$$
  For homogeneous NIMFA (4), the steady-state equation (3) becomes
  11 $$\begin{aligned} \left( \beta A - \delta I \right) v_\infty = \beta {\text {diag}}\left( v_\infty \right) A v_\infty . \end{aligned}$$
  We substitute (11) in (10) and obtain that
  12 $$\begin{aligned} \frac{d c(t)}{d t } v_\infty&\approx \left( c(t) - c^2(t) \right) \left( \beta A - \delta I \right) v_\infty . \end{aligned}$$
  Since $v_\infty \approx \gamma x_1$ around the epidemic threshold, it holds that $A v_\infty \approx \rho (A) v_\infty$. Hence, we obtain that
  13 $$\begin{aligned} \frac{d c(t)}{d t } v_\infty&\approx \left( c(t) - c^2(t) \right) \left( \beta \rho (A) - \delta \right) v_\infty . \end{aligned}$$
  Left-multiplying (13) by $v^T_\infty$ and dividing by $v^T_\infty v_\infty$ yields that
  14 $$\begin{aligned} \frac{d c(t)}{d t }&\approx \left( c(t) - c^2(t) \right) \left( \beta \rho (A) - \delta \right) . \end{aligned}$$
  The *logistic differential equation* (14) has been introduced by Verhulst (1838) as a population growth model and has a closed-form solution.

Due to the two intuitive steps above, NIMFA (4) reduces around the threshold $R_0 \approx 1$ to the one-dimension differential equation (14). Solving (14) for the function *c*(*t*) gives an approximation of the viral state *v*(*t*) by (9). The solution approach is applicable to other dynamics on networks, see for instance (Devriendt and Lambiotte 2020).

However, the reasoning above is not rigorous for two reasons. First, the viral state vector *v*(*t*) is not exactly parallel to the steady state $v_\infty$. To be more specific, instead of (9) it holds that

15 $$\begin{aligned} v(t) = c(t) v_\infty + \xi (t) \end{aligned}$$

for some $N\times 1$ *error vector* $\xi (t)$ which is orthogonal to the steady-state vector $v_\infty$. In Sect. 5.2, we use (15) as an ansatz for solving NIMFA (1).

Second, the steady-state vector $v_\infty$ is not exactly parallel to the principal eigenvector $x_1$. More precisely, we must consider the vector $\eta$ in (6). Since $\eta \ne 0$, the step from (12) to (13) is affected by an error.

### The solution around the epidemic threshold

Based on the motivation in Sect. 5.1, we aim to solve the NIMFA differential equations (1) around the epidemic threshold criterion $R_0 = 1$. The ansatz (15) forms the basis for our solution approach. From the orthogonality of the error vector $\xi (t)$ and the steady-state vector $v_\infty$, it follows that the function *c*(*t*) at time *t* equals

16 $$\begin{aligned} c(t) = \frac{1}{ \Vert v_\infty \Vert ^2_2 } v^T_\infty v(t). \end{aligned}$$

The error vector $\xi (t)$ at time *t* follows from (15) and (16) as

17 $$\begin{aligned} \xi (t) = \left( I - \frac{1}{ \Vert v_\infty \Vert ^2_2 }v_\infty v^T_\infty \right) v(t). \end{aligned}$$

Our solution approach is based on two steps. First, we show that^6^ the error term $\xi (t)$ satisfies $\xi (t)= {\mathcal {O}}((R_0 - 1)^2)$ at every time *t* when $R_0 \downarrow 1$. Hence, the error term $\xi (t)$ converges to zero *uniformly in time* *t*. Second, we find the solution of the scalar function *c*(*t*) at the limit $R_0 \downarrow 1$.

Assumption 2 implies that^7^ the viral state *v*(*t*) does not overshoot the steady-state $v_\infty$:

#### Lemma 1

Under Assumptions 1 to 3, it holds that $v_i(t) \le v_{\infty , i}$ for all nodes *i* at every time $t \ge 0$. Furthermore, it holds that $0 \le c(t) \le 1$ at every time $t \ge 0$.

#### Proof

Appendix C. $$\square $$

Theorem 2 states that the error term $\xi (t)$ converges to zero in the order of $(R_0 - 1)^2$ when $R_0 \downarrow 1$.

#### Theorem 2

Under Assumptions 1 to 3, there exist constants $\sigma _1, \sigma _2 >0$ such that the error term $\xi (t)$ at any time $t\ge 0$ is bounded by

18 $$\begin{aligned} \Vert \xi (t) \Vert _2 \le \Vert \xi (0) \Vert _2 e^{-\sigma _1 t} + \sigma _2 (R_0 - 1)^2 \end{aligned}$$

when the basic reproduction number $R_0$ approaches 1 from above.

#### Proof

Appendix D. $$\square $$

Under Assumption 2, the steady-state $v_\infty$ is exponentially stable for NIMFA in *discrete time* (Prasse and Van Mieghem 2019). If the steady-state $v_\infty$ is exponentially stable, then the error vector $\xi (t)$ goes to zero exponentially fast, since $\xi (t)$ is orthogonal to $v_\infty$. Thus, the first addend on the right-hand side in (18) is rather expectable, under the conjecture that the steady-state $v_\infty$ is exponentially stable also for *continuous-time* NIMFA (1). Regarding this work, the most important implication of Theorem 2 is that $\xi (t) = {\mathcal {O}}\left( (R_0-1)^2\right)$ *uniformly in time* *t* when $R_0 \downarrow 1$, provided the initial value $\xi (0)$ of the error vector is negligibly small.

We define the constant $\varUpsilon (0)$, which depends on the initial viral state *v*(0), as

19 $$\begin{aligned} \varUpsilon (0) = {\mathrm{artanh}} \left( 2 \frac{ v^T_\infty v(0) }{ \Vert v_\infty \Vert ^2_2 } -1 \right) . \end{aligned}$$

Furthermore, we define the *viral slope* *w*, which determines the speed of convergence to the steady-state $v_\infty$, as

$$\begin{aligned} w = (R_0 - 1)\sum ^N_{l=1} \delta _l \left( x_1\right) ^2_l. \end{aligned}$$

Then, building on Theorems 1 and 2, we obtain our main result:

#### Theorem 3

Suppose that Assumptions 1 to 3 hold and that, for some constant $p>1$, $\Vert \xi (0) \Vert _2 = {\mathcal {O}}\left( (R_0 - 1)^p\right)$ when $R_0 \downarrow 1$. Furthermore, define

20 $$\begin{aligned} v_{\mathrm{apx}}(t)= \frac{1}{2} \left( 1 + \tanh \left( \frac{w}{2} t + \varUpsilon (0)\right) \right) v_\infty . \end{aligned}$$

Then, there exists some constant $\sigma >0$ such that

21 $$\begin{aligned} \frac{\Vert v(t) - v_{\mathrm{apx}}(t)\Vert _2}{ \Vert v_\infty \Vert _2} \le&\sigma (R_0 - 1)^{s-1} \quad \forall t\ge 0, \end{aligned}$$

where $s = {\mathrm{min}}\{p, 2\}$, when the basic reproduction number $R_0$ approaches 1 from above.

#### Proof

Appendix E. $$\square $$

We emphasise that Theorem 3 holds for any connected graph corresponding to the infection rate matrix *B*. Theorem 3 is in agreement with the universality of the SIS prevalence (Van Mieghem 2016). The bound (21) states a convergence of the viral state *v*(*t*) to the approximation $v_{\text {apx}}(t)$ which is uniform in time *t*. Furthermore, since both the viral state *v*(*t*) and the approximation $v_{\text {apx}}(t)$ converge to the steady-state $v_\infty$, it holds that $\Vert v(t) - v_{\mathrm{apx}}(t)\Vert _2\rightarrow 0$ when $t \rightarrow \infty$. At time $t=0$, we obtain from Theorem 3 and (17) that

$$\begin{aligned} \Vert v(0) - v_{\mathrm{apx}}(0)\Vert _2 = \Vert \xi (0) \Vert _2. \end{aligned}$$

Since $\Vert \xi (0) \Vert _2 = {\mathcal {O}}\left( (R_0 - 1)^p\right)$ and, by Theorem 1, $\Vert v_\infty \Vert _2 = {\mathcal {O}}\left( R_0 - 1\right)$, we obtain that

$$\begin{aligned} \frac{ \Vert v(0) - v_{\mathrm{apx}}(0)\Vert _2 }{\Vert v_\infty \Vert _2 } = {\mathcal {O}}\left( (R_0 - 1)^{p-1}\right) . \end{aligned}$$

Hence, for general $t\ge 0$ the approximation error $\Vert v(t) - v_{\mathrm{apx}}(t)\Vert _2 / \Vert v_\infty \Vert _2$ does not converge to zero faster than ${\mathcal {O}}\left( (R_0 - 1)^{p-1}\right)$, and the bound (21) is best possible (up to the constant $\sigma$) when $p\le 2$. With (17), the term $\Vert \xi (0) \Vert _2$ in Theorem 2 can be expressed explicitly with respect to the initial viral state *v*(0) and the steady-state $v_\infty$. In particular, it holds that $\Vert \xi (0) \Vert _2 \le \Vert v(0)\Vert _2$. Furthermore, if the initial viral state *v*(0) is parallel to the steady-state vector $v_\infty$, then it holds that $\xi (0)=0$. Thus, if the initial viral state *v*(0) is small or parallel to the steady-state vector $v_\infty$, then it holds that $\xi (0)=0$ and the bound (21) on the approximation error vector becomes

22 $$\begin{aligned} \frac{\Vert v(t) - v_{\mathrm{apx}}(t)\Vert _2}{ \Vert v_\infty \Vert _2} \le \sigma (R_0 - 1 ) \quad \forall t\ge 0. \end{aligned}$$

The time-dependent solution to NIMFA (1) at the epidemic threshold criterion $R_0 = 1$ depends solely on the viral slope *w*, the steady-state vector $v_\infty$ and the initial viral state *v*(0). The viral slope *w* converges to zero as $R_0 \downarrow 1$. Thus, Theorem 3 implies that the convergence time to the steady-state $v_\infty$ goes to infinity when $R_0 \downarrow 1$, even though the steady-state $v_\infty$ converges to zero. More precisely, it holds:

#### Corollary 1

Suppose that Assumptions 1 and 3 hold and that the initial viral state *v*(0) equals $v(0) = r_0 v_\infty$ for some scalar $r_0 \in (0, 1)$. Then, for any scalar $r_1 \in [r_0 , 1)$, the largest time $t_{01}$ at which the viral state satisfies $v_i(t_{01}) \le r_1 v_{\infty , i}$ for every node *i* converges to

$$\begin{aligned} t_{01} = \frac{1}{w} \log \left( \frac{r_1}{r_0} \frac{1 - r_0}{1 - r_1}\right) \end{aligned}$$

when the basic reproduction number $R_0$ approaches 1 from above.

#### Proof

Appendix F. $$\square $$

We combine Theorem 1 and Theorem 3 to obtain Corollary 2.

#### Corollary 2

Suppose that Assumptions 1 to 3 hold and that, for some constant $p>1$, $\Vert \xi (0) \Vert _2 = {\mathcal {O}}\left( (R_0 - 1)^p\right)$ when $R_0 \downarrow 1$. Furthermore, define

23 $$\begin{aligned} {\tilde{v}}_{\mathrm{apx}}(t)= \left( 1 + \tanh \left( \frac{w}{2} t + \varUpsilon (0)\right) \right) \frac{\gamma }{2} x_1. \end{aligned}$$

Then, there exists some constant $\sigma >0$ such that

$$\begin{aligned} \frac{\Vert v(t) - {\tilde{v}}_{\mathrm{apx}}(t)\Vert _2}{ \Vert v_\infty \Vert _2} \le&\sigma (R_0 - 1)^{s-1} \quad \forall t\ge 0, \end{aligned}$$

where $s = {\mathrm{min}}\{p, 2\}$, when the basic reproduction number $R_0$ approaches 1 from above.

In contrast to Theorem 3, the approximation error $\Vert v(t) - {\tilde{v}}_{\mathrm{apx}}(t)\Vert _2$ in Corollary 2 does *not* converge to zero when $t \rightarrow \infty$, since we replaced the steady-state $v_\infty$ by the first-order approximation of Theorem 1. Corollary 2 implies that

24 $$\begin{aligned} \frac{v_i(t)}{v_j(t)} \rightarrow \frac{{\tilde{v}}_{{\text {apx}}, i}(t)}{{\tilde{v}}_{{\text {apx}}, j}(t)} = \frac{(x_1)_i}{(x_1)_j} \end{aligned}$$

*at every time* *t* when $R_0 \downarrow 1$, provided that the initial viral state *v*(0) is small or parallel to the steady-state vector $v_\infty$. From (24) it follows that, around the epidemic threshold criterion $R_0 = 1$, the eigenvector centrality (Van Mieghem 2010) fully determines the “dynamical importance” of node *i* versus node *j*.

For homogeneous NIMFA (4), the infection rate matrix *B* and the curing rate matrix *S* reduce to $B = \beta A$ and $S = \delta I$, respectively. Hence, the effective infection rate matrix becomes $W = \frac{\beta }{\delta } A$, and the principal eigenvector $x_1$ of the effective infection rate matrix *W* equals the principal eigenvector of the adjacency matrix *A*. Furthermore, the limit process $R_0 \downarrow 1$ reduces to $\tau \downarrow \tau _c$, with the *effective infection rate* $\tau = \frac{\beta }{\delta }$ and the *epidemic threshold* $\tau _c = 1/\rho (A)$. For homogeneous NIMFA (4), Theorem 3 reduces to:

#### Corollary 3

Suppose that Assumptions 1 to 3 hold and consider the viral state *v*(*t*) of homogeneous NIMFA (4). Furthermore, suppose that $\Vert \xi (0) \Vert _2 = {\mathcal {O}}\left( (\tau - \tau _c)^p\right)$ for some constant $p>1$ when $\tau \downarrow \tau _c$ and define

25 $$\begin{aligned} v_{\mathrm{apx}}(t)= \frac{1}{2}\left( 1 + \tanh \left( \frac{ ( \tau - \tau _c ) \delta }{2 \tau _c} t + \varUpsilon (0)\right) \right) v_\infty . \end{aligned}$$

Then, there exists some constant $\sigma >0$ such that

$$\begin{aligned} \frac{\Vert v(t) - v_{\mathrm{apx}}(t)\Vert _2}{ \Vert v_\infty \Vert _2} \le&\sigma (\tau - \tau _c)^{s-1} \quad \forall t\ge 0, \end{aligned}$$

where $s = {\mathrm{min}}\{p, 2\}$, when the effective infection rate $\tau$ approaches the epidemic threshold $\tau _c$ from above.

#### Proof

Appendix G. $$\square $$

From Corollary 3, we can obtain the analogue to Corollary 2 for NIMFA (4) with homogeneous spreading parameters $\beta , \delta$. Furthermore, the approximation $v_{\mathrm{apx}}(t)$ defined by (25) equals the exact solution (Van Mieghem 2014b) of homogeneous NIMFA (4) on a regular graph, provided that the initial state $v_i(0)$ is the same for every node *i*. In particular, the *net dose* $\varrho (t)$, a crucial quantity in Van Mieghem (2014b); Kendall (1948), is related to the viral slope *w* via $\varrho (t)=wt$.

Theorem 3 and Corollary 3 suggest that, around the epidemic threshold criterion $R_0 =1$, the dynamics of heterogeneous NIMFA (1) closely resembles the dynamics of homogeneous NIMFA (4). In particular, we pose the question: *Can heterogeneous NIMFA* (1) *be reduced to homogeneous NIMFA* (4) *around the epidemic threshold criterion* $R_0 = 1$ *by choosing the homogeneous spreading parameters* $\beta , \delta$ *and the adjacency matrix* *A* *accordingly?*

#### Theorem 4

Consider heterogeneous NIMFA (1) with given spreading parameters $\beta _{ij}, \delta _i$. Suppose that Assumptions 1 to 3 hold and that, for some constant $p>1$, $\Vert \xi (0) \Vert _2 = {\mathcal {O}}\left( (R_0 - 1)^p\right)$ when the basic reproduction number $R_0$ approaches 1 from above. Define the homogeneous NIMFA system

26 $$\begin{aligned} \frac{d v_{i, {{\mathrm{hom}}}} (t)}{d t }&= - \delta _{{\mathrm{hom}}} v_{i, {{\mathrm{hom}}}}(t) + \beta _{ii, {{\mathrm{hom}}}} \left( 1-v_{i, {{\mathrm{hom}}}}(t)\right) v_{i, {{\mathrm{hom}}}}(t) \nonumber \\&\quad + \left( 1-v_{i, {{\mathrm{hom}}}}(t)\right) \beta _{{\mathrm{hom}}} \sum ^N_{j=1, j\ne i} v_{j, {{\mathrm{hom}}}}(t), \end{aligned}$$

where the homogeneous curing rate $\delta _{{\mathrm{hom}}}$ equals

27 $$\begin{aligned} \delta _{{\mathrm{hom}}} = \frac{\sum ^N_{l=1} \delta _l \left( x_1\right) ^3_l}{\sum ^N_{l=1} \left( x_1\right) ^3_l}, \end{aligned}$$

the homogeneous infection rate $\beta _{{\mathrm{hom}}}$ equals

28 $$\begin{aligned} \beta _{{\mathrm{hom}}}= \frac{\delta _{{\mathrm{hom}}}}{\sum ^N_{l=1} \left( x_1\right) _l } \left( 1 + \gamma \sum ^N_{l=1} \left( x_1\right) ^3_l \right) \underset{l=1,\ldots , N}{{\text {min}}} \left( x_1\right) _l \end{aligned}$$

with the variable $\gamma$ defined by (7), and the self-infection rates $\beta _{ii, {{\mathrm{hom}}}}$ equal

$$\begin{aligned} \beta _{ii,{{\mathrm{hom}}}} = \beta _{{\mathrm{hom}}} \left( \frac{1}{\underset{l=1,\ldots , N}{{\text {min}}} \left( x_1\right) _l } - \frac{1}{\left( x_1\right) _i} \right) \sum ^N_{j=1} \left( x_1\right) _j + \beta _{{\mathrm{hom}}}. \end{aligned}$$

Then, if $v_{{\mathrm{hom}}}(0)=v(0)$, there exists some constant $\sigma >0$ such that

$$\begin{aligned} \frac{\Vert v(t) - v_{{\mathrm{hom}}}(t)\Vert _2}{ \Vert v_\infty \Vert _2} \le&\sigma (R_0 - 1)^{s-1} \quad \forall t\ge 0, \end{aligned}$$

where $s = {\mathrm{min}}\{p, 2\}$, when the basic reproduction number $R_0$ approaches 1 from above.

#### Proof

Appendix H. $$\square $$

In other words, when $R_0 \downarrow 1$, for any contact network and any spreading parameters $\delta _i, \beta _{ij}$, heterogeneous NIMFA (1) can be reduced to homogeneous NIMFA (4) on a complete graph plus self-infection rates $\beta _{ii, {{\mathrm{hom}}}}$. We emphasise that the sole influence of the topology on the viral spread is given by the self-infection rates $\beta _{ii, {{\mathrm{hom}}}}$. *Thus, under Assumptions* 1*to* 3*, the network topology has a surprisingly small impact on the viral spread around the epidemic threshold.*

## Numerical evaluation

We are interested in evaluating the accuracy of the closed-form expression $v_{\text {apx}}(t)$, given by (20), when the basic reproduction number $R_0$ is close, but not equal, to one. We generate an adjacency matrix *A* according to different random graph models. If $a_{ij}= 1$, then we set the infection rates $\beta _{ij}$ to a uniformly distributed random number in [0.4, 0.6] and, if $a_{ij}= 0$, then we set $\beta _{ij}=0$. We set the *initial curing rates* $\delta ^{(0)}_l$ to a uniformly distributed random number in [0.4, 0.6]. To set the basic reproduction number $R_0$, we set the curing rates $\delta _l$ to a multiple of the initial curing rates $\delta ^{(0)}_l$, i.e. $\delta _l = \sigma \delta ^{(0)}_l$ for every node *l* and some scalar $\sigma$ such that $\rho (W) = R_0$. Thus, we realise the limit process $R_0 \downarrow 1$ by changing the scalar $\sigma$. Only in Sect. 6.2, we consider homogeneous spreading parameters by setting $\beta _{ij}=0.5$ and $\delta ^{(0)}_i=0.5$ for all nodes *i*, *j*. Numerically, we obtain the “exact” NIMFA viral state sequence *v*(*t*) by Euler’s method for discretisation, i.e.,

29 $$\begin{aligned} \left. \frac{d v_i (t )}{d t }\right| _{t = Tk} \approx \frac{ v_i(Tk) - v_i\left( T(k-1)\right) }{T} \end{aligned}$$

for a small sampling time *T* and a discrete time slot $k\in {\mathbb {N}}$. In Prasse and Van Mieghem (2019), we derived an upper bound $T_{\text {max}}$ on the sampling time *T* which ensures that the discretisation (29) of NIMFA (1) converges to the steady-state $v_\infty$. We set the sampling time *T* to $T = T_{\text {max}}/100$. Except for Sect. 6.3, we set the initial viral state to $v(0)= 0.01 v_\infty$. We define the convergence time $t_{\text {conv}}$ as the smallest time *t* at which

$$\begin{aligned} \left| v_i(t_{\text {conv}}) - v_{\infty , i} \right| \le 0.01 \end{aligned}$$

holds for every node *i*. Thus, at the convergence time $t_{\text {conv}}$ the viral state $v(t_{\text {conv}})$ has practically converged to the steady-state $v_\infty$. We evaluate Theorem 3 with respect to the approximation error $\epsilon _V$, which we define as

$$\begin{aligned} \epsilon _V = \frac{1}{N t_{\text {conv}}} \sum ^N_{i=1} \int ^{t_{\text {conv}}}_0 \frac{\left| v_i\left( {\tilde{t}}\right) - v_{{\text {apx}}, i}\left( {\tilde{t}}\right) \right| }{v_{\infty , i}} d{\tilde{t}}. \end{aligned}$$

All results are averaged over 100 randomly generated networks.

### Approximation accuracy around the epidemic threshold

**Fig. 1:**
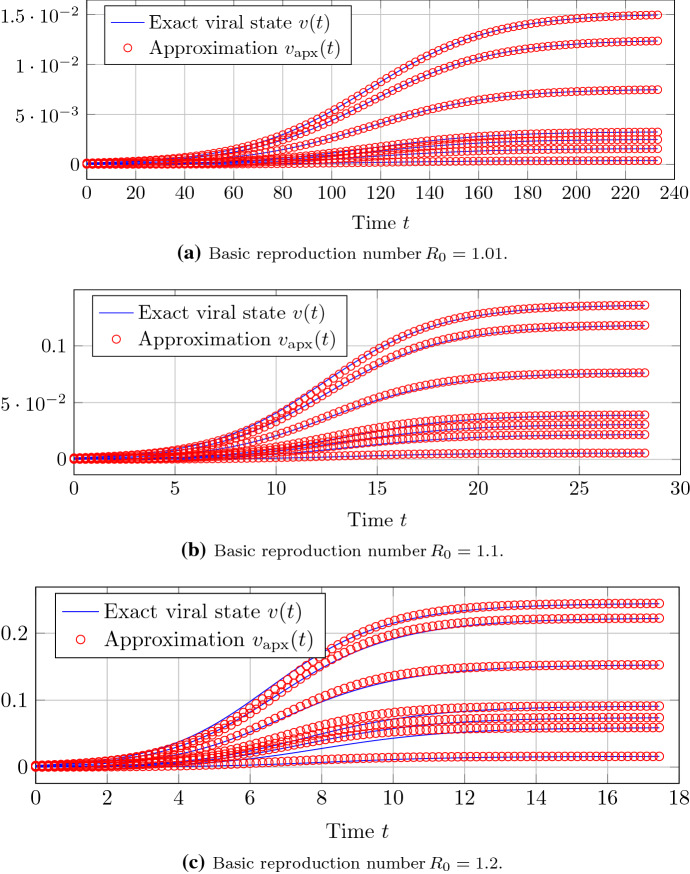
For a Barabási–Albert random graph with $N=500$ nodes, the approximation accuracy of Theorem 3 is depicted. Each of the sub-plots shows the viral state traces $v_i(t)$ of seven different nodes *i*, including the node *i* with the greatest steady-state $v_{\infty , i}$

We generate a Barabási–Albert random graph (Barabási and Albert 1999) with $N=500$ nodes and the parameters $m_0 = 5$, $m= 2$. Figure 1 gives an impression of the accuracy of the approximation of Theorem 3 around the epidemic threshold criterion $R_0 = 1$. For a basic reproduction number $R_0 \le 1.1$, the difference of the closed-form expression of Theorem 3 to the exact NIMFA viral state trace is negligible.

**Fig. 2:**
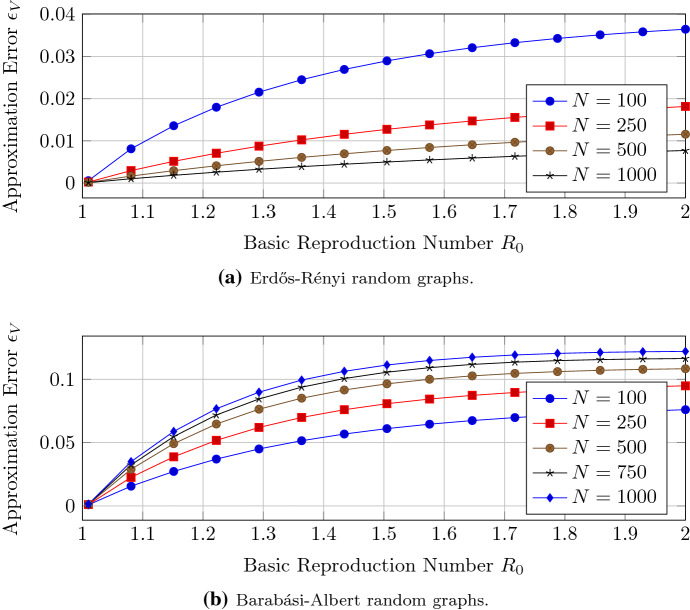
The approximation error $\epsilon _V$ of the NIMFA solution versus the basic reproduction number $R_0$ for different network sizes *N*

We aim for a better understanding of the accuracy of the closed-form expression of Theorem 3 when the basic reproduction number $R_0$ converges to one. We generate Barabási–Albert and Erdős–Rényi connected random graphs with $N=100,\ldots , 1000$ nodes. The link probability of the Erdős–Rényi graphs (Erdős and Rényi 1960) is set to $p_{\text {ER}}=0.05$. Figure 2 illustrates the convergence of the approximation of Theorem 3 to the exact solution of NIMFA (1). Around the threshold criterion $R_0=1$, the approximation error $\epsilon _V$ converges linearly to zero with respect to the basic reproduction number $R_0$, which is in agreement with Theorem 3. The greater the network size *N*, the greater is the approximation error $\epsilon _V$ for Barabási–Albert networks. The greater the network size *N*, the lower is the approximation error $\epsilon _V$ for Erdős–Rényi graphs.

### Impact of degree heterogeneity on the approximation accuracy

**Fig. 3:**
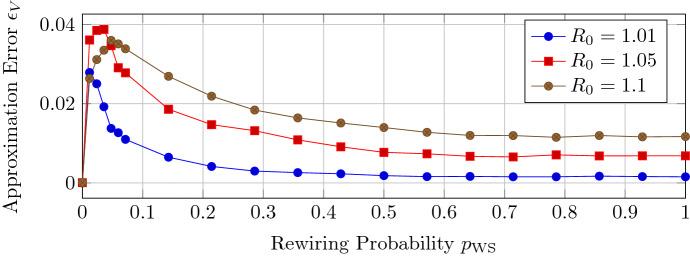
The approximation error $\epsilon _V$ versus the link rewiring probability $p_{\text {WS}}$ for Watts–Strogatz random graphs with $N=100$ nodes and homogeneous spreading parameters $\beta , \delta$

For NIMFA (4) with homogeneous spreading parameters $\beta , \delta$, the approximation $v_{\text {apx}}(t)$ defined by (4) is exact if the contact network is a regular graph. We are interested how the approximation accuracy changes with respect to the heterogeneity of the node degrees. We generate Watts–Strogatz (Watts and Strogatz 1998) random graphs with $N=100$ nodes and an average node degree of 4. We vary the link rewiring probability $p_{\text {WS}}$ from $p_{\text {WS}}=0$, which correspond to a regular graph, to $p_{\text {WS}}= 1$, which corresponds to a “completely random” graph. Figure 3 depicts the approximation error $\epsilon _V$ versus the rewiring probability $p_{\text {WS}}$ for homogeneous spreading parameters $\beta , \delta$. Interestingly, the approximation error reaches a maximum and improves when the adjacency matrix *A* is more random.

### Impact of general initial viral states on the approximation accuracy

**Fig. 4:**
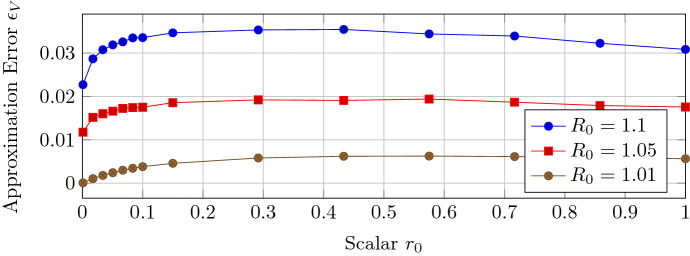
The approximation error $\epsilon _V$ versus the scalar $r_0$, which controls the variance of the randomly generated initial viral state *v*(0), for Barabási–Albert networks with $N= 250$ nodes

**Fig. 5:**
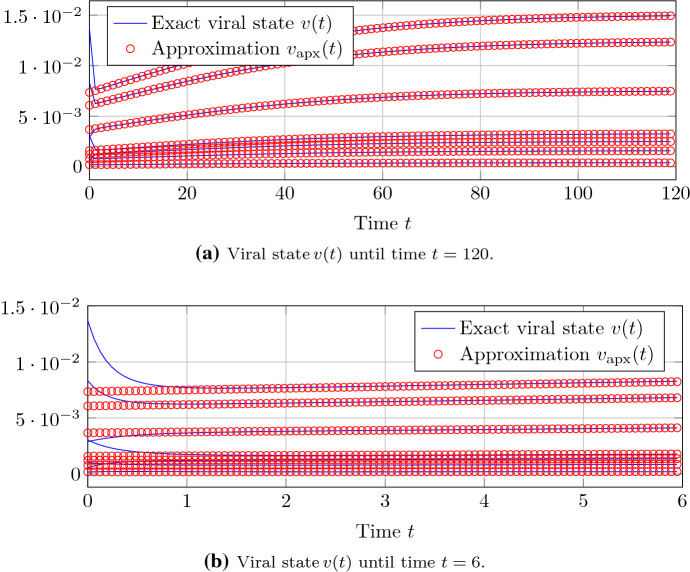
For a Barabási–Albert random graph with $N=500$ nodes, a basic reproduction number $R_0=1.01$ and a randomly generated initial viral state *v*(0), the approximation accuracy of Theorem 3 is depicted. The viral state traces $v_i(t)$ of seven different nodes *i* are depicted

Theorem 3 required that the initial error $\xi (0)$ converges to zero, which means that the initial viral state *v*(0) must be parallel to the steady-state $v_\infty$ or, since $\Vert \xi (0)\Vert _2 \le \Vert v(0)\Vert$, converge to zero. To investigate whether the approximation of Theorem 3 is accurate also when the initial error $\xi (0)$ does not converge to zero, we set the initial viral state $v_i(0)$ of every node *i* to a uniformly distributed random number in $(0, r_0 v_{\infty , i}]$ for some scalar $r_0 \in (0, 1]$. By increasing the scalar $r_0$, the initial viral state *v*(0) is “more random”. Figure 4 shows that the approximation error $\epsilon _V$ is almost unaffected by an initial viral state *v*(0) that is neither parallel to the steady-state $v_\infty$ nor small. Figure 5 shows that the viral state *v*(*t*) converges rapidly to the approximation $v_{\text {apx}}(t)$ as time *t* increases.

For general initial viral states *v*(0) with $\xi (0)\ne 0$, it holds that $v_{\text {apx}}(0) \ne v(0)$ since the approximation $v_{\text {apx}}(0)$ is parallel to the steady-state vector $v_\infty$. Hence, the approximation $v_{\text {apx}}(t)$ does not converge *point-wise* to the viral state *v*(*t*) when $R_0 \downarrow 1$. However, based on the results shown in Figs. 4 and 5, we conjecture *convergence with respect to the* $L_2$*-norm* for general initial viral states *v*(0) when $R_0 \downarrow 1$.

#### Conjecture 1

Suppose that Assumptions 1 to 3 hold. Then, it holds for the approximation $v_{\mathrm{apx}}(t)$ defined by (20) that

$$\begin{aligned} \frac{1}{\Vert v_\infty \Vert _2 } \int ^\infty _0 \Vert v(t) - v_{\mathrm{apx}}(t)\Vert _2 dt \rightarrow 0 \end{aligned}$$

when the basic reproduction number $R_0$ approaches 1 from above.

### Directed infection rate matrix

**Fig. 6:**
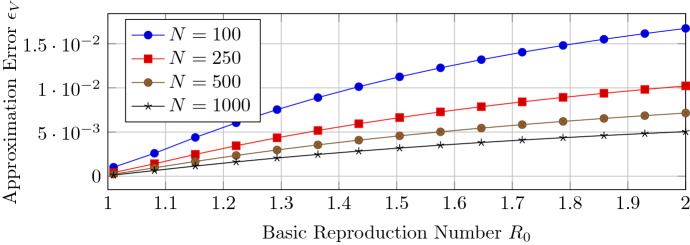
The approximation error $\epsilon _V$ of the NIMFA solution versus the basic reproduction number $R_0$ for *directed* Erdős–Rényi graphs for different network sizes *N*

The proof of Theorem 3 relies on a symmetric infection rate matrix *B* as stated by Assumption 3. We perform the same numerical evaluation as shown in Fig. 2 in Sect. 6.1 with the only difference that we generate strongly connected *directed* Erdős–Rényi random graphs. Figure 6 demonstrates the accuracy of the approximation $v_{\text {apx}}(t)$ for a directed infection rate matrix *B*, which leads us to:

#### Conjecture 2

Suppose that Assumptions 1 and 2 hold and that the infection rate matrix *B* is irreducible but, in contrast to Assumption 3, not necessarily symmetric. Then, the viral state *v*(*t*) is “accurately described” by the approximation $v_{\mathrm{apx}}(t)$ when the basic reproduction number $R_0$ approaches 1 from above.

**Fig. 7:**
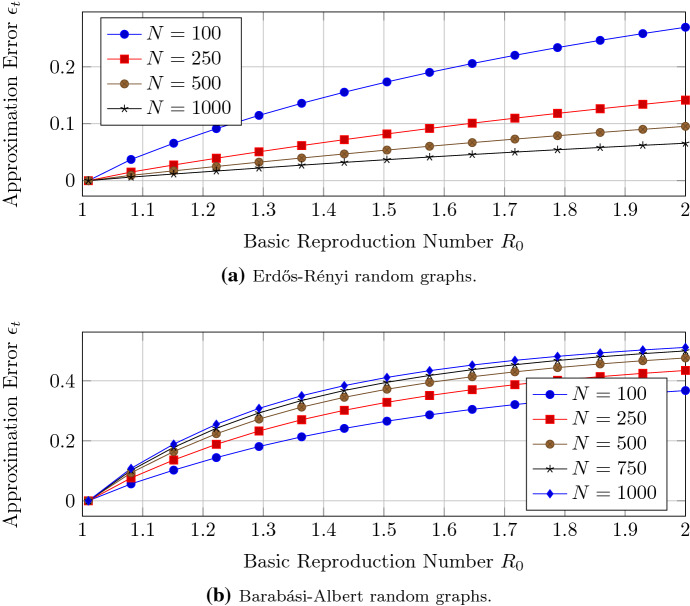
The approximation error $\epsilon _t$ of the convergence time $t_{01}$ versus the basic reproduction number $R_0$ for different network sizes *N*

### Accuracy of the approximation of the convergence time

Corollary 1 gives the expression of the convergence time $t_{01}$ from the initial viral state $v(0) = r_0 v_\infty$ to the viral state $v(t_{01}) \le r_1 v_\infty$ for any scalars $0< r_0 \le r_1 <1$ around the epidemic threshold criterion $R_0 = 1$. We set the scalars to $r_0 = 0.01$ and $r_1= 0.9$ and define the approximation error

$$\begin{aligned} \epsilon _t = \frac{\left| {\hat{t}}_{01} - t_{01} \right| }{t_{01}}, \end{aligned}$$

where $t_{01}$ denotes the exact convergence time and ${\hat{t}}_{01}$ denotes the approximate expression of Corollary 1. We generate Barabási–Albert and Erdős–Rényi random graphs with $N=100,\ldots , 1000$ nodes. Figure 7 shows that Corollary 1 gives an accurate approximation of the convergence time $t_{01}$ when the basic reproduction number $R_0$ is reasonably close to one.

### Reduction to a complete graph with homogeneous spreading parameters

**Fig. 8:**
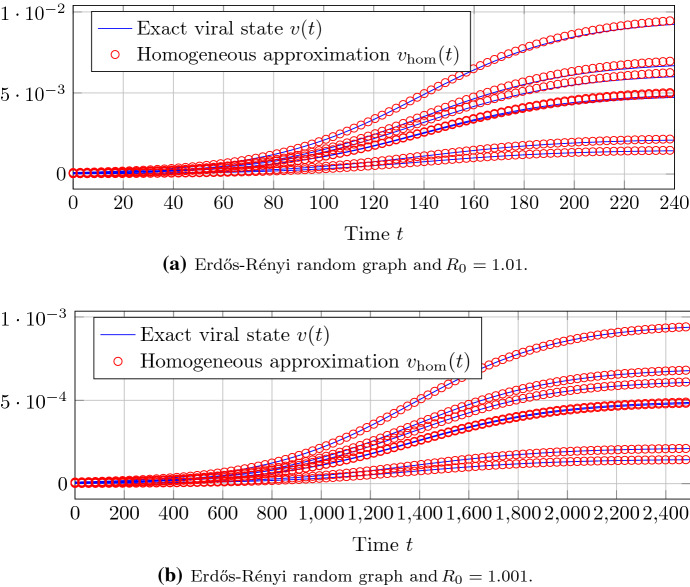
The approximation accuracy of Theorem 4 on a Erdős–Rényi random graph with $N=100$ nodes. Each of the sub-plots shows the viral state traces $v_i(t)$ of seven different nodes *i*, including the node *i* with the greatest steady-state $v_{\infty , i}$

**Fig. 9:**
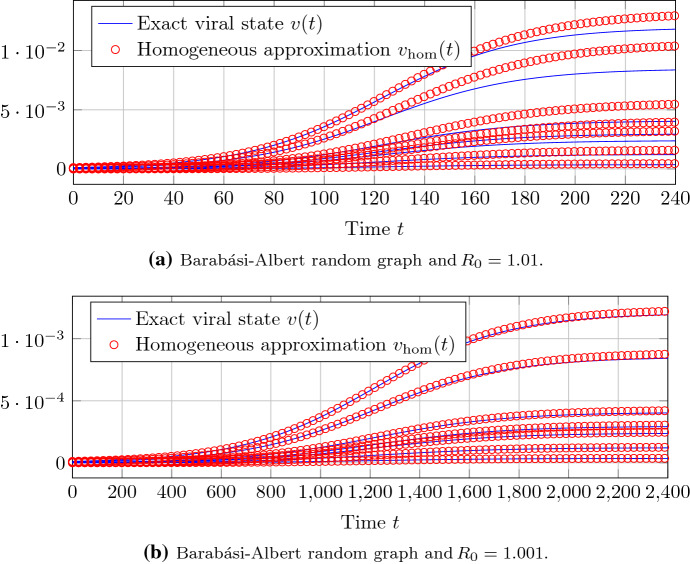
The approximation accuracy of Theorem 4 on a Barabási–Albert random graph with $N=100$ nodes. Each of the sub-plots shows the viral state traces $v_i(t)$ of seven different nodes *i*, including the node *i* with the greatest steady-state $v_{\infty , i}$

Theorem 4 states that, around the epidemic threshold, heterogeneous NIMFA (1) on any graph can be reduced to homogeneous NIMFA (4) on a complete graph. Figures 8 and 9 show the approximation accuracy of Theorem 4 for Erdős–Rényi and Barabási–Albert random graphs, respectively. To accurately approximate heterogeneous NIMFA on Barabási–Albert graphs by homogeneous NIMFA on a complete graph, the basic reproduction number $R_0$ must be closer to 1 than for Erdős–Rényi graphs.

## Conclusion

We solved the NIMFA governing equations (1) with heterogeneous spreading parameters around the epidemic threshold when the initial viral state *v*(0) is small or parallel to the steady-state $v_\infty$, provided that the infection rates are symmetric ($\beta _{ij}=\beta _{ji}$). Numerical simulations demonstrate the accuracy of the solution when the basic reproduction number $R_0$ is close, but not equal, to one. Furthermore, the solution serves as an accurate approximation also when the initial viral state *v*(0) is neither small nor parallel to the steady-state $v_\infty$. We observe four important implications of the solution of NIMFA around the epidemic threshold.

First, the viral state *v*(*t*) is almost parallel to the steady-state $v_\infty$ for every time $t\ge 0$. On the one hand, since the viral dynamics approximately remain in a one-dimensional subspace of ${\mathbb {R}}^N$, an accurate network reconstruction is numerically not viable around the epidemic threshold (Prasse and Van Mieghem 2018). Furthermore, when the basic reproduction number $R_0$ is large, then the viral state *v*(*t*) rapidly converges to the steady-state $v_\infty$, which, again, prevents an accurate network reconstruction. On the other hand, only the principal eigenvector $x_1$ of the effective infection rate matrix *W* and the viral slope *w* are required to predict the viral state dynamics around the epidemic threshold. *Thus, around the epidemic threshold, the prediction of an epidemic does not require an accurate network reconstruction.*

Second, the eigenvector centrality (with respect to the principal eigenvector $x_1$ of the effective infection rate matrix *W*) gives a complete description of the dynamical importance of a node *i* around the epidemic threshold. *In particular, the ratio* $v_i(t)/v_j(t)$ *of the viral states of two nodes* *i*, *j* *does not change over time* *t*.

Third, around the epidemic threshold, we gave an expression of the convergence time $t_{01}$ to approach the steady-state $v_\infty$. The viral state *v*(*t*) converges to the steady-state $v_\infty$ exponentially fast. *However, as the basic reproduction number* $R_0$ *approaches one, the convergence time* $t_{01}$ *goes to infinity.*

Fourth, around the epidemic threshold, NIMFA with heterogeneous spreading parameter on any graph can be reduced to NIMFA with homogeneous spreading parameters on the complete graph plus self-infection rates.

Potential generalisations of the solution of NIMFA to non-symmetric infection rate matrices *B* or time-dependent spreading parameters $\beta _{ij}(t), \delta _l(t)$ stand on the agenda of future research.

## Nomenclature

The eigenvalues of the effective infection rate matrix *W* are denoted, in decreasing order, by $| \lambda _1 | \ge \cdots \ge |\lambda _N|$. The principal eigenvector of unit length of the matrix *W* is denoted by $x_1$ and satisfies $W x_1 = \lambda _1 x_1$. The greatest and smallest curing rate in $\{\delta _1,\ldots , \delta _N\}$ are denoted by $\delta _{\text {max}}$ and $\delta _{\text {min}}$, respectively. The numerical radius *r*(*M*) for an $N \times N$ matrix *M* is defined as (Horn and Johnson 1990)

30 $$\begin{aligned} r(M) = \underset{z \in {\mathbb {C}}^N}{{\text {max}}} ~ \left| \frac{z^H M z}{z^H z}\right| , \end{aligned}$$

where $z^H$ is the conjugate transpose of a complex $N\times 1$ vector *z*. For a square matrix *M*, we denote the 2-norm by $\Vert M \Vert _2$, which equals the largest singular value of *M*. In particular, it holds that the 2-norm of the curing rate matrix *S* equals $\Vert S \Vert _2 = \delta _{\text {max}}$. Table 1 summarises the nomenclature.

**Table 1:** Nomenclature

$\beta _{ij}$	Infection rate from node *j* to node *i*
*B*	Infection rate matrix; $B_{ij} = \beta _{ij}$
*c*(*t*)	Projection of the viral state *v*(*t*) on the steady-state $v_\infty$; see (16)
$\delta _i$	Curing rate of node *i*
${\text {diag}}(x)$	$N \times N$ diagonal matrix with $x\in {\mathbb {R}}^N$ on its diagonal
*I*	$N \times N$ identity matrix
$\lambda _k$	*k*-th eigenvalue of the matrix *W*; $\lambda _1 > \lambda _2 \ge \cdots \ge \lambda _N$
*N*	Number of nodes
$\Vert M \Vert _2$	2-norm of a matrix *M*: largest singular value of *M*
*r*(*M*)	Numerical radius of a square matrix *M*; see (30)
$R_0$	Basic reproduction number; $R_0 = \rho (W)=\lambda _1$
$\rho (M)$	Spectral radius of a square matrix *M*
*S*	Curing rate matrix; $S = {\text {diag}}(\delta _1,\ldots , \delta _N)$
*u*	$N\times 1$ all-one vector $u = (1,\ldots , 1)^T$
*v*(*t*)	$N\times 1$ viral state vector *v*(*t*) at time $t \ge 0$; $v_i(t) \in [0, 1]$ for $i=1,\ldots , N$
$v_\infty$	Steady-state vector, see Definition 2
*w*	The viral slope; $w = (R_0 - 1)\sum ^N_{l=1} \delta _l \left( x_1\right) ^2_l$
*W*	Effective infection rate matrix $W = S^{-1}B$; $\rho (W) > 1$
${\tilde{W}}$	Symmetric $N\times N$ matrix ${\tilde{W}} = S^{-\frac{1}{2}} B S^{-\frac{1}{2}}$
$x_k$	*k*-th eigenvector of the matrix *W*; $W x_k = \lambda _k x_k$
$\xi (t)$	Viral state component that is orthogonal to $v_\infty$; $\xi (t) = v(t) - c(t)v_\infty$

## Proof of Theorem 1

The steady-state $v_\infty$ solely depends on the effective infection rate matrix *W*: By left-multiplication of (3) with the diagonal matrix $S^{-1}$, we obtain that

31 $$\begin{aligned} \left( W - I \right) v_\infty = {\text {diag}}\left( v_\infty \right) W v_\infty . \end{aligned}$$

In general, the effective infection rate matrix *W*, defined in (5) as $W=S^{-1}B$, is asymmetric, which prevents a straightforward adaptation of the proof in Van Mieghem (2012, Lemma 4). However, the matrix *W* is similar to the matrix

32 $$\begin{aligned} {\tilde{W}}&= S^{-\frac{1}{2}}B S^{-\frac{1}{2}} \nonumber \\&= S^{\frac{1}{2}} W S^{-\frac{1}{2}}. \end{aligned}$$

Since the infection rate matrix *B* is symmetric under Assumption 3, the matrix ${\tilde{W}}$ is symmetric. Hence, the matrix ${\tilde{W}}$, and also the effective infection rate matrix *W*, are diagonalisable. With (32), we write the steady-state (31) with respect to the symmetric matrix ${\tilde{W}}$ as

33 $$\begin{aligned} \left( {\tilde{W}} - I \right) S^{\frac{1}{2}} v_\infty = {\text {diag}}\left( v_\infty \right) {\tilde{W}} S^{\frac{1}{2}} v_\infty . \end{aligned}$$

We decompose the matrix ${\tilde{W}}$ as

34 $$\begin{aligned} {\tilde{W}} = \lambda _1 {\tilde{x}}^T_1 {\tilde{x}}_1 + \sum ^N_{k=2} \lambda _k {\tilde{x}}^T_k {\tilde{x}}_k, \end{aligned}$$

where the eigenvalues of ${\tilde{W}}$ are real and equal to $\lambda _1 > \lambda _2 \ge \cdots \ge \lambda _N$ with the corresponding normalized eigenvectors denoted by ${\tilde{x}}_1,\ldots , {\tilde{x}}_N$. Then, the steady-state vector $v_\infty$ can be expressed as linear combination

$$\begin{aligned} v_\infty = \sum ^N_{l = 1} \psi _l {\tilde{x}}_l, \end{aligned}$$

where the coefficients equal $\psi _l = v^T_\infty {\tilde{x}}_l$. To prove Theorem 1, we would like to express the coefficients $\psi _1,\ldots , \psi _N$ as a power series around $R_0 = 1$. However, in the limit process $\left( B, S\right) \rightarrow \left( B^*, S^*\right)$, the eigenvectors ${\tilde{x}}_1,\ldots , {\tilde{x}}_N$ of the matrix ${\tilde{W}}$ are not necessarily constant. Hence, the coefficients $\psi _l$ depend on the full matrix ${\tilde{W}}$ and not only on the basic reproduction number $R_0$. To overcome the challenge of non-constant eigenvectors ${\tilde{x}}_1,\ldots , {\tilde{x}}_N$ in the limit process $\left( B, S\right) \rightarrow \left( B^*, S^*\right)$, we define the symmetric auxiliary matrix

35 $$\begin{aligned} M(z) = z {\tilde{x}}^T_1 {\tilde{x}}_1 + \sum ^N_{k=2} \lambda _k {\tilde{x}}^T_k {\tilde{x}}_k ~ \in {\mathbb {R}}^{N \times N} \end{aligned}$$

for a scalar $z \ge 1$. Thus, the matrix *M*(*z*) is obtained from the matrix ${\tilde{W}}$ by replacing the largest eigenvalue $\lambda _1$ of ${\tilde{W}}$ by *z*. In particular, the definition of the matrix *M*(*z*) in (35) and (34) illustrate that $M(\lambda _1) = {\tilde{W}}$. When the matrix ${\tilde{W}}$ is formally replaced by the matrix *M*(*z*), the steady-state equation (33) becomes

36 $$\begin{aligned} \left( M(z) - I \right) S^{\frac{1}{2}} {\tilde{v}}(z) = {\mathrm{diag}}\left( {\tilde{v}}(z)\right) M(z) S^{\frac{1}{2}} {\tilde{v}}(z) \end{aligned}$$

where the $N\times 1$ vector ${\tilde{v}}(z)$ denotes the solution of (36). Since $M(R_0) = {\tilde{W}}$, the solution of (36) at $z=R_0$ and the solution to (33) coincide, i.e., ${\tilde{v}}(R_0)=v_\infty$. Lemma 2 expresses the solution of the equation (36) as a power series.

### Lemma 2

Suppose that Assumptions 1 and 3 hold. If (*B*, *S*) is sufficiently close to $(B^*, S^*)$, then the $N\times 1$ vector ${\tilde{v}}(z)$ which satisfies (36) equals

37 $$\begin{aligned} {\tilde{v}}(z) = (z - 1) \left( \sum ^N_{l=1} \frac{1}{\sqrt{\delta _l}}\left( {\tilde{x}}_1\right) ^3_l\right) ^{-1} S^{-\frac{1}{2}} {\tilde{x}}_1 + \phi (z), \end{aligned}$$

where the $N\times 1$ vector $\phi (z)$ satisfies $\Vert \phi (z) \Vert _2 \le \sigma (B, S) (z - 1)^2$ for some scalar $\sigma (B, S)$ when *z* approaches 1 from above.

### Proof

The proof is an adaptation of the proof (Van Mieghem 2012, Lemma 4). We express the solution ${\tilde{v}}(z)$ of (36) as linear combination of the vectors $S^{-\frac{1}{2}}{\tilde{x}}_1,\ldots , S^{-\frac{1}{2}}{\tilde{x}}_N$, i.e.,

38 $$\begin{aligned} {\tilde{v}}(z) = \sum ^N_{k = 1} \psi _k(z) S^{-\frac{1}{2}} {\tilde{x}}_k. \end{aligned}$$

Since the diagonal matrix $S^{-\frac{1}{2}}$ is full rank, the vectors $\left( S^{-\frac{1}{2}} {\tilde{x}}_k\right)$, where $k=1,\ldots , N$, are linearly independent. Furthermore, we express the coefficients $\psi _k(z)$ as a power series

39 $$\begin{aligned} \psi _k(z) = \sum ^\infty _{j=0} g_j(k) \left( z - 1\right) ^j, \end{aligned}$$

where $g_0(k) = 0$ for every $k=1,\ldots , N$, since (Lajmanovich and Yorke 1976) it holds that ${\tilde{v}}(z) = 0$ when $z = 1$. We denote the eigenvalues of the matrix *M*(*z*) by

40 $$\begin{aligned} \lambda _k(z)= {\left\{ \begin{array}{ll} z \quad &{}\quad \text {if } k = 1,\\ \lambda _k &{}\quad \text {if } k \ge 2.\end{array}\right. } \end{aligned}$$

By substituting (38) into (36), we obtain that

$$\begin{aligned} \sum ^N_{k = 1} \left( \lambda _k(z) - 1\right) \psi _k(z) {\tilde{x}}_k = {\text {diag}}\left( \sum ^N_{l = 1} \psi _l(z) {\tilde{x}}_l \right) S^{-\frac{1}{2}}\sum ^N_{k = 1} \lambda _k(z) \psi _k(z) {\tilde{x}}_k \end{aligned}$$

and left-multiplying with the eigenvector ${\tilde{x}}^T_m$, for any $m=1,\ldots , N$, yields

41 $$\begin{aligned} \left( \lambda _m(z) - 1\right) \psi _m(z)&= \sum ^N_{n = 1} \left( {\tilde{x}}_m\right) _n \sum ^N_{l=1} \psi _l(z) \left( {\tilde{x}}_l\right) _n \frac{1}{\sqrt{\delta _n}} \sum ^N_{k=1} \psi _k(z) \lambda _k(z) \left( {\tilde{x}}_k\right) _n. \end{aligned}$$

We define

$$\begin{aligned} X(m, l, k) = \sum ^N_{n = 1} \frac{1}{\sqrt{\delta _n}} \left( {\tilde{x}}_m\right) _n \left( {\tilde{x}}_l\right) _n \left( {\tilde{x}}_k\right) _n. \end{aligned}$$

Then, we rewrite (41) as

42 $$\begin{aligned} \left( \lambda _m(z) - 1\right) \psi _m(z)&= \sum ^N_{l=1} \sum ^N_{k=1} \psi _l(z) \psi _k(z) \lambda _k(z) X(m, l, k). \end{aligned}$$

First, we focus on the left-hand side of (42), which we denote by

$$\begin{aligned} \theta _m (z) = \left( \lambda _m(z) - 1\right) \psi _m(z). \end{aligned}$$

With the power series (39), we obtain that

$$\begin{aligned} \theta _m (z) = \left( \lambda _m(z) - 1\right) \sum ^\infty _{j=1} g_j(m) \left( z - 1\right) ^j . \end{aligned}$$

Further rewriting yields that

43 $$\begin{aligned} \theta _m (z)&=\left( \lambda _m(z) - z + z - 1\right) \sum ^\infty _{j=1} g_j(m) \left( z - 1\right) ^j \nonumber \\&= \left( \lambda _m(z) - z \right) \sum ^\infty _{j=1} g_j(m) \left( z - 1\right) ^j + \sum ^\infty _{j=1} g_j(m) \left( z - 1\right) ^{j+1} \nonumber \\&= \sum ^\infty _{j=1} \left( \lambda _m(z) - z \right) g_j(m) \left( z - 1\right) ^j + \sum ^\infty _{j=2} g_{j-1}(m) \left( z - 1\right) ^j. \end{aligned}$$

Second, we rearrange the right-hand side of (42) as

$$\begin{aligned} \theta _m (z)&= \lambda _1(z)\sum ^N_{l=1} \psi _l(z) \psi _1(z) X(m, l, 1) + \sum ^N_{l=1} \sum ^N_{k=2} \psi _l(z) \psi _k(z) \lambda _k(z) X(m, l, k). \end{aligned}$$

By the definition of $\lambda _k(z)$ in (40) it holds that $\lambda _1(z) = z$, and we obtain that

44 $$\begin{aligned} \theta _m (z) =&(z - 1)\sum ^N_{l=1} \psi _l(z) \psi _1(z) X(m, l, 1) + \sum ^N_{l=1} \sum ^N_{k=1} \psi _l(z) \psi _k(z) {\tilde{\lambda }}_k X(m, l, k), \end{aligned}$$

where

$$\begin{aligned} {\tilde{\lambda }}_k ={\left\{ \begin{array}{ll} 1 &{}\quad \text {if } k = 1,\\ \lambda _k &{}\quad \text {if } k \ge 2. \end{array}\right. } \end{aligned}$$

Introducing the power series (39) into (44) and executing the Cauchy product for $\psi _l(z) \psi _k(z)$ yields

$$\begin{aligned} \theta _m (z)&= \sum ^\infty _{j=1}\left( \sum ^{j-1}_{n=1}\sum ^N_{l=1} g_{j-n}(1) g_n(l) X(m, l, 1)\right) \left( z - 1\right) ^{j+1}\\&\quad +\sum ^\infty _{j=1} \left( \sum ^{j-1}_{n=1}\sum ^N_{l=1}\sum ^N_{k=1} g_{j-n}(l) g_n(k) {\tilde{\lambda }}_k X(m, l, k) \right) \left( z - 1\right) ^j. \end{aligned}$$

We shift the index *j* in the first term and obtain

45 $$\begin{aligned} \theta _m (z)&= \sum ^\infty _{j=2}\left( \sum ^{j-2}_{n=1}\sum ^N_{l=1} g_{j-1-n}(1) g_n(l) X(m, l, 1)\right) \left( z - 1\right) ^{j}\nonumber \\&\quad +\sum ^\infty _{j=1} \left( \sum ^{j-1}_{n=1}\sum ^N_{l=1}\sum ^N_{k=1} g_{j-n}(l) g_n(k) {\tilde{\lambda }}_k X(m, l, k) \right) \left( z - 1\right) ^j. \end{aligned}$$

Finally, we equate powers in $(z-1)^j$ in (43) and (45), which yields for $j=1$ that

46 $$\begin{aligned} \left( \lambda _m(z) -z\right) g_1(m) = 0 \end{aligned}$$

for every $m=1,\ldots , N$. The spectral radius of the limit $W^*$ of the effective infection rate matrix *W* equals 1. Furthermore, the limit $W^*$ is a non-negative and irreducible matrix. Thus, the eigenvalues of the limit $W^*$ obey $\lambda ^*_1 = 1 > |\lambda ^*_m|$ for every $m\ge 2$, which implies that $|\lambda _m| < 1$ for every $m\ge 2$ provided that (*B*, *S*) is sufficiently close to $(B^*, S^*)$. With the definition of $\lambda _m(z)$ in (40), we obtain from (46) that $g_1(m) = 0$ when $m \ge 2$ provided that (*B*, *S*) is sufficiently close to $(B^*, S^*)$, since $z\ge 1$.

For $j\ge 2$, equating powers in (45) yields that

47 $$\begin{aligned} \left( \lambda _m(z) - z \right) g_j(m) + g_{j-1}(m)&= \sum ^{j-2}_{n=1}\sum ^N_{l=1} g_{j-1-n}(1) g_n(l) X(m, l, 1) \nonumber \\&\quad + \sum ^{j-1}_{n=1}\sum ^N_{l=1}\sum ^N_{k=1} g_{j-n}(l) g_n(k) {\tilde{\lambda }}_k X(m, l, k). \end{aligned}$$

In particular, for the case $j=2$, we obtain

48 $$\begin{aligned} \left( \lambda _m(z) - z \right) g_2(m) + g_{1}(m)&= \sum ^N_{l=1}\sum ^N_{k=1} g_{1}(l) g_1(k) {\tilde{\lambda }}_k X(m, l, k) \nonumber \\&= g_{1}(1) g_1(1) X(m, 1, 1), \end{aligned}$$

since $g_1(l)=0$ for all $l\ge 2$ and ${\tilde{\lambda }}_1 = 1$. Since $\lambda _1(z) = z$, we obtain for $m=1$ from (48) that

$$\begin{aligned} g_{1}(1)&= g_1(1)^2 X(1, 1, 1) \end{aligned}$$

and, hence,

$$\begin{aligned} g_{1}(1)&= \frac{1}{X(1, 1, 1)} = \left( \sum ^N_{l = 1} \frac{1}{\sqrt{\delta _l}} \left( {\tilde{x}}_1\right) ^3_l\right) ^{-1}. \end{aligned}$$

Since $g_1(m) = 0$ for $m\ge 2$, we obtain that the power series (38) for the solution ${\tilde{v}}(z)$ of (36) becomes

49 $$\begin{aligned} {\tilde{v}}(z) = ( z - 1 ) g_{1}(1) S^{-\frac{1}{2}} {\tilde{x}}_1 + \phi (z), \end{aligned}$$

where the $N\times 1$ vector $\phi (z)$ equals

$$\begin{aligned} \phi (z) = \sum ^N_{k = 1} \left( \sum ^\infty _{j=2} g_j(k) \left( z - 1\right) ^j\right) S^{-\frac{1}{2}} {\tilde{x}}_k. \end{aligned}$$

Thus, it holds $\Vert \phi (z)\Vert _2 = {\mathcal {O}}\left( (z-1)^2\right)$ when *z* approaches 1 from above, which proves Lemma 2. $\square$

We believe that, based on (47), a recursion for the coefficients $g_j(k)$ can be obtained for powers $j\ge 2$, similar to the proof of Van Mieghem (2012, Lemma 4). The radius of convergence of the power series (49) is an open problem, see also He and Van Mieghem (2020). To express the solution ${\tilde{v}}(z)$ in (37) in terms of the principal eigenvector $x_1$ of the effective infection rate matrix *W*, we propose Lemma 3.

### Lemma 3

Under Assumptions 1 and 3, it holds that

50 $$\begin{aligned} \left( \sum ^N_{l=1} \frac{1}{\sqrt{\delta _l}}\left( {\tilde{x}}_1\right) ^3_l\right) ^{-1} S^{-\frac{1}{2}} {\tilde{x}}_1 =\frac{\sum ^N_{l=1} \delta _l \left( x_1\right) ^2_l}{\sum ^N_{l=1} \delta _l \left( x_1\right) ^3_l} x_1. \end{aligned}$$

### Proof

From (32), it follows that the principal eigenvector ${\tilde{x}}_1$ of the matrix ${\tilde{W}}$ and the principal eigenvector $x_1$ of the effective infection rate matrix *W* are related via

$$\begin{aligned} {\tilde{x}}_1 = \frac{1}{\left\Vert S^{\frac{1}{2}} x_1 \right\Vert _2 } S^{\frac{1}{2}} x_1, \end{aligned}$$

or, component-wise,

$$\begin{aligned} ({\tilde{x}}_1)_l = \frac{1}{\left\Vert S^{\frac{1}{2}} x_1 \right\Vert _2 } \sqrt{\delta _l} ~ (x_1)_l. \end{aligned}$$

Then, we rewrite the left-hand side of (50) as

$$\begin{aligned} \left( \sum ^N_{l=1} \frac{1}{\sqrt{\delta _l}}\left( {\tilde{x}}_1\right) ^3_l\right) ^{-1} S^{-\frac{1}{2}} {\tilde{x}}_1 =\left( \sum ^N_{l=1} \delta _l \left( x_1\right) ^3_l\right) ^{-1} \left\Vert S^{\frac{1}{2}} x_1 \right\Vert ^2_2 x_1, \end{aligned}$$

which simplifies to

$$\begin{aligned} \left( \sum ^N_{l=1} \frac{1}{\sqrt{\delta _l}}\left( {\tilde{x}}_1\right) ^3_l\right) ^{-1} S^{-\frac{1}{2}} {\tilde{x}}_1 = \frac{x^T_1 S x_1 }{\sum ^N_{l=1} \delta _l \left( x_1\right) ^3_l} x_1. \end{aligned}$$

Writing out the quadratic form in the numerator completes the proof. $\square$

The basic reproduction number $R_0$ converges to 1 when $(B, S) \rightarrow (B^*, S^*)$. Hence, if (*B*, *S*) is sufficiently close to $(B^*, S^*)$, then the basic reproduction number $R_0$ is smaller than the radius of convergence of the power series (38). Thus, if (*B*, *S*) is sufficiently close to $(B^*, S^*)$, then the solution ${\tilde{v}}(R_0)$ to (36) at $z=R_0$ follows with Lemma 2 as

$$\begin{aligned} {\tilde{v}}(R_0)&= (R_0 - 1) \left( \sum ^N_{l=1} \frac{1}{\sqrt{\delta _l}}\left( {\tilde{x}}_1\right) ^3_l\right) ^{-1} S^{-\frac{1}{2}} {\tilde{x}}_1 + \phi (R_0) \nonumber \\&=\gamma x_1 + \phi (R_0), \end{aligned}$$

where the last equality follows from Lemma 3 and the definition of the scalar $\gamma$ in (7). We emphasise that Lemma 2 implies that $\gamma = {\mathcal {O}}(R_0 -1)$ and, hence, $\Vert {\tilde{v}}(R_0) \Vert _2= {\mathcal {O}}(R_0 -1)$ as $(B, S) \rightarrow (B^*, S^*)$. Since $M(R_0) = {\tilde{W}}$, the solution of (36) at $z=R_0$ and the solution to (33) coincide, i.e., ${\tilde{v}}(R_0)=v_\infty$. Thus, from the definition of the vector $\eta$ in (6), we obtain that

51 $$\begin{aligned} \left\Vert \eta \right\Vert _2&= \left\Vert v_\infty - \gamma x_1 \right\Vert _2 \nonumber \\&=\left\Vert \phi (R_0) \right\Vert _2 \end{aligned}$$

when $(B, S) \rightarrow (B^*, S^*)$. Lemma 2 states that $\left\Vert \phi (z) \right\Vert _2 = {\mathcal {O}}\left( (z-1)^2\right)$ as $z \downarrow 1$. Hence, we obtain from (51) that

52 $$\begin{aligned} \left\Vert \eta \right\Vert _2 \le \sigma (B, S) (R_0 - 1)^2 \end{aligned}$$

for some scalar $\sigma (B, S)$ when $(B, S) \rightarrow (B^*, S^*)$.

Furthermore, when (*B*, *S*) converge to the limit $(B^*, S^*)$, the scalar $\sigma (B, S)$ converges to some limit $\sigma (B^*, S^*)$. Hence, by defining the constant

$$\begin{aligned} \sigma = \sigma (B^*, S^*) + \epsilon _\sigma \end{aligned}$$

for some $\epsilon _\sigma > 0$, it holds that

$$\begin{aligned} \sigma (B, S) < \sigma , \end{aligned}$$

for all (*B*, *S*) which are sufficiently close to $(B^*, S^*)$. Finally, we obtain from (52) that

$$\begin{aligned} \left\Vert \eta \right\Vert _2 \le \sigma (R_0 - 1)^2 \end{aligned}$$

when (*B*, *S*) approaches $$(B^*, S^*)$$.

## Proof of Lemma 1

We divide Lemma 1 into two parts. In Sect. C.1, we prove that the viral state *v*(*t*) does not overshoot the steady-state $v_\infty$. In Sect. C.2, we show that the function *c*(*t*) lies in the interval [0, 1].

### Absence of overshoot

The proof follows the same reasoning as Prasse and Van Mieghem (2019, Corollary 1). Assume that at some time $t_0$ it holds $v_i(t_0) = v_{\infty , i}$ for some node *i* and that $v_j(t_0) \le v_{\infty , j}$ for every node *j*. Since $v_i (t_0) = v_{\infty , i}$, the NIMFA equation (1) yields that

$$\begin{aligned} \left. \frac{d v_i (t)}{d t }\right| _{t = t_0} = - \delta _i v_{\infty , i} + (1 - v_{\infty , i})\sum ^N_{j = 1} \beta _{i j} v_j(t_0). \end{aligned}$$

Since $v_j(t_0) \le v_{\infty , j}$ for every node *j*, we obtain that

$$\begin{aligned} \left. \frac{d v_i (t)}{d t }\right| _{t = t_0} \le - \delta _i v_{\infty , i} + (1 - v_{\infty , i})\sum ^N_{j = 1} \beta _{i j} v_{\infty , j}=0, \end{aligned}$$

where the last equality follows from the steady-state equation (3). Thus, $v_i (t_0) = v_{\infty , i}$ implies that $\left. \frac{d v_i (t)}{d t } \right| _{t = t_0} \le 0$, which means that, at time $t_0$, the viral state $v_i(t_0)$ does not increase. Hence, the viral state $v_i(t_0)$ cannot exceed the steady-state $v_{\infty , i}$ at any time $t \ge 0$.

### Boundedness of the function *c*(*t*)

Relation (16) indicates that

53 $$\begin{aligned} c(t) = \frac{1}{\Vert v_\infty \Vert ^2_2} v^T_\infty v(t)=\frac{1}{\Vert v_\infty \Vert ^2_2} \left( v_{\infty , 1} v_1(t) + \cdots + v_{\infty , N} v_N(t) \right) \end{aligned}$$

Section C.1 shows that Assumption 2 implies that $v_i(t)\le v_{\infty , i}$ for all nodes *i* and every time *t*. Thus, we obtain from (53) that

$$\begin{aligned} c(t) \le \frac{1}{\Vert v_\infty \Vert ^2_2} \left( v_{\infty , 1} v_{\infty , 1} + \cdots + v_{\infty , N} v_{\infty , N} \right) = 1 \end{aligned}$$

Analogously, since $v_i(t)\ge 0$ for all nodes *i* and every time *t*, we obtain from (53) that $c(t) \ge 0$.

## Proof of Theorem 2

By inserting the ansatz (15) into the NIMFA equations (2), we obtain that

54 $$\begin{aligned} \frac{d c(t)}{d t } v_\infty + \frac{d \xi (t)}{d t }&= \varLambda _1(t) + \varLambda _2(t). \end{aligned}$$

Here, the function $\varLambda _1(t)$ is given by

$$\begin{aligned} \varLambda _1(t) = \left( B - S\right) c(t)v_\infty - c^2(t) {\text {diag}}(v_\infty )B v_\infty , \end{aligned}$$

which simplifies, with the steady-state equation (3), to

55 $$\begin{aligned} \varLambda _1(t) = \left( c(t) - c^2(t) \right) \left( B - S \right) v_\infty . \end{aligned}$$

The function $\varLambda _2(t)$ is given by

$$\begin{aligned} \varLambda _2(t)&= \left( B - S \right) \xi (t) - c(t) {\text {diag}}(\xi (t)) B v_\infty - c(t){\text {diag}}(v_\infty ) B \xi (t) \\&\quad - {\text {diag}}(\xi (t)) B \xi (t). \end{aligned}$$

With ${\text {diag}}(\xi (t)) B v_\infty = {\text {diag}}(B v_\infty ) \xi (t)$, we obtain that

56 $$\begin{aligned} \varLambda _2(t)&= \left( B - S - c(t){\text {diag}}(B v_\infty ) - c(t){\text {diag}}(v_\infty )B \right) \xi (t) \nonumber \\&\quad - {\text {diag}}(\xi (t)) B \xi (t). \end{aligned}$$

To show that the error term $\xi (t)$ converges to zero at every time *t* when $\left( B, S\right) \rightarrow \left( B^*, S^*\right)$, we consider the squared Euclidean norm $\Vert \xi (t)\Vert ^2_2$. The convergence of the squared norm $\Vert \xi (t)\Vert ^2_2$ to zero implies the convergence of the error term $\xi (t)$ to zero. The derivative of the squared norm $\Vert \xi (t)\Vert ^2_2$ is given by

$$\begin{aligned} \frac{d \Vert \xi (t)\Vert ^2_2}{d t } = 2 \xi ^T(t) \frac{d \xi (t)}{d t }. \end{aligned}$$

Thus, we obtain from (54) that

57 $$\begin{aligned} \frac{1}{2} \frac{d \Vert \xi (t)\Vert ^2_2}{d t } = \xi ^T(t) \varLambda _1(t)+ \xi ^T(t)\varLambda _2(t), \end{aligned}$$

since $\xi ^T(t) v_\infty =0$ by definition of $\xi (t)$. We do not know how to solve (57) exactly, and we resort to bounding the two addends on the right-hand side of (57) in Sects. D.1 and D.2, respectively. In Sect. D.3 we complete the proof of Theorem 2 by deriving an upper bound on the squared norm $\Vert \xi (t)\Vert ^2_2$.

### Upper bound on $\xi ^T(t) \varLambda _1(t)$

We obtain an upper bound on the projection of the function $\varLambda _1(t)$ onto the error vector $\xi (t)$, which is linear with respect to the norm $\Vert \xi (t)\Vert _2$:

#### Lemma 4

Under Assumptions 1 to 3, it holds at every time $t\ge 0$ that

$$\begin{aligned} \left| \xi ^T(t) \varLambda _1(t) \right| \le \frac{1}{4} \delta _{\mathrm{max}}\left( \gamma ( R_0 - 1 ) + (R_0 + 1) \Vert \eta \Vert _2 \right) \Vert \xi (t)\Vert _2 . \end{aligned}$$

#### Proof

From (55) and the definition of the matrix *W* in (5) it follows that

$$\begin{aligned} \xi ^T(t) \varLambda _1(t) = \left( c(t) - c^2(t) \right) \xi ^T(t) S \left( W - I\right) v_\infty . \end{aligned}$$

With Theorem 1, we obtain

$$\begin{aligned} \xi ^T(t) \varLambda _1(t) = \left( c(t) - c^2(t) \right) \left( \gamma ( R_0 - 1 ) \xi ^T(t) S x_1 + \xi ^T(t) S (W - I)\eta \right) . \end{aligned}$$

The triangle inequality yields that

58 $$\begin{aligned} \left| \xi ^T(t) \varLambda _1(t) \right| \le \left| c(t) - c^2(t) \right| \left( | \gamma ( R_0 - 1 )| \left| \xi ^T(t) S x_1\right| + \left| \xi ^T(t) S (W - I)\eta \right| \right) . \end{aligned}$$

With the Cauchy–Schwarz inequality, the first addend in (58) is upper-bounded by

$$\begin{aligned} \left| \xi ^T(t) S x_1\right|&\le \Vert S^T \xi (t)\Vert _2 \Vert x_1 \Vert _2\\&=\Vert S \xi (t)\Vert _2, \end{aligned}$$

since $\Vert x_1 \Vert _2 = 1$ and the matrix *S* is symmetric. The matrix 2-norm is sub-multiplicative, which yields that

$$\begin{aligned} \left| \xi ^T(t) S x_1\right|&\le \Vert S \Vert _2 \Vert \xi (t)\Vert _2 \\&= \delta _{\text {max}} \Vert \xi (t)\Vert _2. \end{aligned}$$

Thus, (58) gives that

59 $$\begin{aligned} \left| \xi ^T(t) \varLambda _1(t) \right| \le \left| c(t) - c^2(t) \right| \left( \gamma ( R_0 - 1 ) \delta _{\text {max}} \Vert \xi (t)\Vert _2 + \left| \xi ^T(t) S (W - I)\eta \right| \right) , \end{aligned}$$

since $\gamma > 0$ and $R_0 >1$. We consider the second addend in (59), which we write with (32) as

$$\begin{aligned} \left| \xi ^T(t) S (W - I)\eta \right| = \left| \xi ^T(t) S^{\frac{1}{2}} ({\tilde{W}} - I)S^{\frac{1}{2}}\eta \right| . \end{aligned}$$

From the Cauchy–Schwarz inequality and the sub-multiplicativity of the matrix norm we obtain

$$\begin{aligned} \left| \xi ^T(t) S (W - I)\eta \right| \le \Vert \xi (t)\Vert _2 \Vert S^{\frac{1}{2}} \Vert _2 \Vert {\tilde{W}} - I \Vert _2 \Vert S^{\frac{1}{2}} \Vert _2 \Vert \eta \Vert _2 . \end{aligned}$$

The triangle inequality and the symmetry of the matrix ${\tilde{W}}$ imply that

$$\begin{aligned} \Vert {\tilde{W}} - I \Vert _2 \le \Vert {\tilde{W}} \Vert _2 + \Vert I \Vert _2 = R_0 + 1. \end{aligned}$$

Thus, we can upper bound the second added in (59) by

$$\begin{aligned} \left| \xi ^T(t) S (W - I)\eta \right| \le \delta _{\text {max}} (R_0 + 1) \Vert \xi (t)\Vert _2 \Vert \eta \Vert _2, \end{aligned}$$

since $\Vert S^{\frac{1}{2}} \Vert _2 = \sqrt{\delta _{\text {max}}}$. Hence, (59) yields the upper bound

$$\begin{aligned} \left| \xi ^T(t) \varLambda _1(t)\right| \le \left| c(t) - c^2(t) \right| \delta _{\text {max}}\left( \gamma ( R_0 - 1 ) + (R_0 + 1) \Vert \eta \Vert _2 \right) \Vert \xi (t)\Vert _2 . \end{aligned}$$

Finally, Lemma 1 states that $0\le c(t)\le 1$, which implies that

$$\begin{aligned} \left| c(t) - c^2(t) \right| \le 1/4 \end{aligned}$$

and completes the proof. $\square$

### Upper bound on $\xi ^T(t) \varLambda _2(t)$

Lemma 5 states an intermediate result, which we will use to bound the projection of the function $\varLambda _2(t)$ onto the error vector $\xi (t)$.

#### Lemma 5

Suppose that Assumptions 1 to 3 hold. Then, at every time $t \ge 0$, it holds that

$$\begin{aligned} \xi ^T(t)\varLambda _2(t) \le - \Vert S^{\frac{1}{2}} \xi (t) \Vert ^2_2 + \xi ^T(t) {\mathrm{diag}}(u - c(t)v_\infty ) B \xi (t). \end{aligned}$$

#### Proof

From (56) it follows that

60 $$\begin{aligned} \xi ^T(t) \varLambda _2(t)&= \xi ^T(t) \left( B - S - c(t){\text {diag}}(B v_\infty ) - c(t){\text {diag}}(v_\infty )B \right) \xi (t) \nonumber \\&\quad -\xi ^T(t) {\text {diag}}(\xi (t)) B \xi (t). \end{aligned}$$

To simplify (60), we aim to bound the last addend of (60) by an expression that is quadratic in the error vector $\xi (t)$. The last addend equals

61 $$\begin{aligned} -\xi ^T(t) {\text {diag}}(\xi (t)) B \xi (t) = \sum ^N_{l=1} \xi ^2_l(t) \sum ^N_{j=1} \beta _{lj} \left( - \xi _j(t) \right) . \end{aligned}$$

Since $v(t) = c(t) v_\infty + \xi (t)$ and $v_i(t) \ge 0$ for every node *i* at every time *t*, it holds that

62 $$\begin{aligned} - \xi _i(t) \le c(t) v_{\infty , i}, \quad i=1,\ldots , N. \end{aligned}$$

By inserting (62) in (61), the last addend of (60) is upper bounded by

$$\begin{aligned} -\xi ^T(t) {\text {diag}}(\xi (t)) B \xi (t) \le \sum ^N_{l=1} \xi ^2_l(t) \sum ^N_{j=1} \beta _{lj} c(t) v_{\infty , j}, \end{aligned}$$

which simplifies to

63 $$\begin{aligned} -\xi ^T(t) {\text {diag}}(\xi (t)) B \xi (t) \le c(t) \xi ^T(t){\text {diag}}(B v_\infty ) \xi (t). \end{aligned}$$

By applying the upper bound (63) to (60), we obtain that

$$\begin{aligned} \xi ^T(t) \varLambda _2(t) \le&\xi ^T(t)\left( B - S - c(t){\text {diag}}(v_\infty )B \right) \xi (t). \end{aligned}$$

With the definition of the matrix ${\tilde{W}}$ in (32), we obtain

$$\begin{aligned} \xi ^T(t) \varLambda _2(t) \le&\xi ^T(t)S^{\frac{1}{2}}\left( {\tilde{W}} - I - c(t){\text {diag}}(v_\infty ) {\tilde{W}} \right) S^{\frac{1}{2}} \xi (t), \end{aligned}$$

and further rearranging completes the proof. $\square$

For any scalar $\varsigma \in [0, 1]$ and any vector $\upsilon \in {\mathbb {R}}^N$, we define

$$\begin{aligned} \varTheta ( \varsigma , \upsilon , B, S ) = \frac{\left| \upsilon ^T {\mathrm{diag}}(u - \varsigma v_\infty ) B \upsilon \right| }{ \Vert S^{\frac{1}{2}} \upsilon \Vert ^2_2}. \end{aligned}$$

Then, we obtain from Lemma 5 that

64 $$\begin{aligned} \xi ^T(t)\varLambda _2(t) \le \left( \varTheta ( c(t), \xi (t), B, S ) - 1 \right) \Vert S^{\frac{1}{2}} \xi (t) \Vert ^2_2. \end{aligned}$$

To upper-bound the term $\varTheta ( c(t), \xi (t), B, S )$, we make use of (parts of) the results of Issos (1966), which are analogues of the Perron–Frobenius Theorem for the numerical radius of a non-negative, irreducible matrix:

#### Theorem 5

(Issos 1966) Let *M* be a real irreducible and non-negative $N\times N$ matrix. Then, there is a positive vector $z \in {\mathbb {R}}^N$ of length $z^T z = 1$ such that $z^T M z = r(M)$. Furthermore, if ${\tilde{z}}^T M {\tilde{z}} = r(M)$ holds for a vector ${\tilde{z}} \in {\mathbb {R}}^N$ of length ${\tilde{z}}^T {\tilde{z}} = 1$, then either ${\tilde{z}} = z$ or ${\tilde{z}} = -z$.

We refer the reader to Issos (1966), Maroulas et al. (2002) and Li et al. (2002) for further results on the numerical radius of non-negative matrices. We apply Theorem 5 to obtain:

#### Lemma 6

Denote the set of $N\times 1$ vectors with at least one positive and at least one negative component as

$$\begin{aligned} {\mathcal {S}} = \left\{ \upsilon \in {\mathbb {R}}^N | \exists i,j: \upsilon _j> 0 > \upsilon _i \right\} . \end{aligned}$$

Then, it holds $\varTheta ( \varsigma , \upsilon , B, S ) < R_0$ for every scalar $\varsigma \in [0,1]$ and for every vector $\upsilon \in {\mathcal {S}}$.

#### Proof

By introducing the $N\times 1$ vector

$$\begin{aligned} {\tilde{\upsilon }} = S^{\frac{1}{2}} \upsilon \end{aligned}$$

and by using (32), we rewrite the term $\varTheta ( \varsigma , \upsilon , B, S )$ as

65 $$\begin{aligned} \varTheta ( \varsigma , \upsilon , B, S ) = \frac{\left| {\tilde{\upsilon }}^T {\text {diag}}(u - \varsigma v_\infty ) {\tilde{W}} {\tilde{\upsilon }} \right| }{\Vert {\tilde{\upsilon }} \Vert ^2_2}. \end{aligned}$$

For every scalar $\varsigma \in [0,1]$ the matrix $({\text {diag}}(u - \varsigma v_\infty ) {\tilde{W}})$ is irreducible and non-negative. Since $\upsilon \in {\mathcal {S}}$ and the matrix *S* is a diagonal matrix with non-negative entries, it holds that ${\tilde{\upsilon }}_i < 0$ and ${\tilde{\upsilon }}_j>0$ for some *i*, *j*. Hence, at least two components of the vector ${\tilde{\upsilon }}$ have different signs, and Theorem 5 implies that (65) is upper-bounded by

$$\begin{aligned} \varTheta ( \varsigma , \upsilon , B, S ) < r\left( {\text {diag}}(u - \varsigma v_\infty ) {\tilde{W}} \right) . \end{aligned}$$

Since the matrix ${\tilde{W}}$ is irreducible and ${\text {diag}}(u - \varsigma v_\infty ) {\tilde{W}} \le {\tilde{W}}$ for every $\varsigma \in [0,1]$, where the inequality holds element-wise, it holds (Li et al. 2002, Corollary 3.6.) that

$$\begin{aligned} \varTheta ( \varsigma , \upsilon , B, S ) < r\left( {\tilde{W}} \right) . \end{aligned}$$

The matrix ${\tilde{W}}$ is symmetric, and, hence, the numerical radius $r\left( {\tilde{W}} \right)$ equals the spectral radius $\rho \left( {\tilde{W}} \right) = R_0$, which yields that

$$\begin{aligned} \varTheta ( \varsigma , \upsilon , B, S ) < R_0. \end{aligned}$$

$$\square $$

Finally, we obtain a bound on the projection of the function $\varLambda _2(t)$ onto the error vector $\xi (t)$:

#### Lemma 7

Under Assumptions 1 to 3, there is some constant $\omega > 0$ such that

$$\begin{aligned} \xi ^T(t)\varLambda _2(t) \le - \omega \delta _{\mathrm{max}} \Vert \xi (t) \Vert ^2_2 \end{aligned}$$

holds at every time $t\ge 0$ when $\left( B, S\right)$ approaches $\left( B^*, S^*\right)$.

#### Proof

We denote the maximum of the function $\varTheta ( \varsigma , \upsilon , B, S )$ with respect to $\varsigma \in [0, 1]$ and $\upsilon \in {\mathcal {S}}$ by

66 $$\begin{aligned} \varTheta _{\text {max}}( B, S ) = \underset{\varsigma \in [0,1], \upsilon \in {\mathcal {S}}}{\text {max}} \quad \varTheta ( \varsigma , \upsilon , B, S ). \end{aligned}$$

As a first step, we consider the value of $\varTheta _{\text {max}}( B^*, S^* )$ at the limit $(B^*, S^*)$. Since the steady-state $v_\infty$ equals to zero at the limit $(B^*, S^*)$, we obtain from (65) that

67 $$\begin{aligned} \varTheta ( \varsigma , \upsilon , B^*, S^* ) = \frac{1}{\Vert {\tilde{\upsilon }} \Vert ^2_2}\left| {\tilde{\upsilon }}^T {\tilde{W}}^* {\tilde{\upsilon }} \right| , \end{aligned}$$

where we denote ${\tilde{W}}^* = \left( S^*\right) ^{-\frac{1}{2}} B^* \left( S^*\right) ^{-\frac{1}{2}}$. Since it holds $R_0 =1$ at the limit $(B^*, S^*)$, Lemma 6 implies that

68 $$\begin{aligned} \varTheta _{\text {max}}( B^*, S^* ) < 1. \end{aligned}$$

As a second step, we consider that the infection rate matrix *B* and the curing rate matrix *S* do not equal the respective limit $B^*$ and $S^*$. Thus, there are non-zero $N\times N$ matrices $\varDelta B, \varDelta S$ and $\varDelta {\tilde{W}}$ such that $B = B^* +\varDelta B$, $S = S^* + \varDelta S$, and ${\tilde{W}} = {\tilde{W}}^* + \varDelta {\tilde{W}}$. Then, we obtain from (65) that

$$\begin{aligned} \varTheta ( \varsigma , \upsilon , B, S ) = \frac{ 1 }{\Vert {\tilde{\upsilon }} \Vert ^2_2} \left| {\tilde{\upsilon }}^T \left( {\tilde{W}}^* - \varsigma {\text {diag}}(v_\infty ) {\tilde{W}}^* + {\text {diag}}(u - \varsigma v_\infty ) \varDelta {\tilde{W}} \right) {\tilde{\upsilon }} \right| , \end{aligned}$$

which is upper-bounded by

69 $$\begin{aligned} \varTheta ( \varsigma , \upsilon , B, S )&\le \frac{ 1 }{\Vert {\tilde{\upsilon }} \Vert ^2_2} \left| {\tilde{\upsilon }}^T {\tilde{W}}^* {\tilde{\upsilon }} \right| + \frac{ 1 }{\Vert {\tilde{\upsilon }} \Vert ^2_2}\left| {\tilde{\upsilon }}^T \varsigma {\text {diag}}(v_\infty ) {\tilde{W}}^* {\tilde{\upsilon }} \right| \nonumber \\&\quad + \frac{ 1 }{\Vert {\tilde{\upsilon }} \Vert ^2_2} \left| {\tilde{\upsilon }}^T {\text {diag}}(u - \varsigma v_\infty ) \varDelta {\tilde{W}} {\tilde{\upsilon }} \right| . \end{aligned}$$

Maximising every addend in (69) independently yields an upper bound on $\varTheta _{\text {max}}( B, S )$ as

70 $$\begin{aligned} \varTheta _{\text {max}}( B, S )&\le \underset{\varsigma \in [0,1], \upsilon \in {\mathcal {S}}}{\text {max}} ~ \frac{1}{\Vert {\tilde{\upsilon }} \Vert ^2_2} \left| {\tilde{\upsilon }}^T {\tilde{W}}^* {\tilde{\upsilon }} \right| \nonumber \\&\quad + \underset{\varsigma \in [0,1], \upsilon \in {\mathcal {S}}}{\text {max}} ~ \frac{1}{\Vert {\tilde{\upsilon }} \Vert ^2_2} \left| {\tilde{\upsilon }}^T \varsigma {\text {diag}}(v_\infty ) {\tilde{W}}^* {\tilde{\upsilon }} \right| \nonumber \\&\quad +\underset{\varsigma \in [0,1], \upsilon \in {\mathcal {S}}}{\text {max}} ~\frac{1}{\Vert {\tilde{\upsilon }} \Vert ^2_2} \left| {\tilde{\upsilon }}^T {\text {diag}}(u - \varsigma v_\infty ) \varDelta {\tilde{W}} {\tilde{\upsilon }} \right| . \end{aligned}$$

In the following, we state upper bounds for each of the three addends in (67) separately. With (67), we write the first addend in (70) as

71 $$\begin{aligned} \underset{\varsigma \in [0,1], \upsilon \in {\mathcal {S}}}{\text {max}} ~ \frac{1}{\Vert {\tilde{\upsilon }} \Vert ^2_2} \left| {\tilde{\upsilon }}^T {\tilde{W}}^* {\tilde{\upsilon }} \right|&= \underset{\varsigma \in [0,1], \upsilon \in {\mathcal {S}}}{\text {max}} \varTheta ( \varsigma , \upsilon , B^*, S^*)\nonumber \\&= \varTheta _{\text {max}}( B^*, S^* ), \end{aligned}$$

where the last equality follows from the definition of $\varTheta _{\text {max}}( B^*, S^* )$ in (66). Regarding the second addend in (70), it holds that

$$\begin{aligned} \underset{\varsigma \in [0,1], \upsilon \in {\mathcal {S}}}{\text {max}} ~ \frac{1}{\Vert {\tilde{\upsilon }} \Vert ^2_2} \left| {\tilde{\upsilon }}^T \varsigma {\text {diag}}(v_\infty ) {\tilde{W}}^* {\tilde{\upsilon }} \right|&\le \underset{\varsigma \in [0,1]}{\text {max}} ~ \underset{\upsilon \in {\mathbb {R}}^N}{\text {max}} ~ \frac{1}{\Vert {\tilde{\upsilon }} \Vert ^2_2} \left| {\tilde{\upsilon }}^T \varsigma {\text {diag}}(v_\infty ) {\tilde{W}}^* {\tilde{\upsilon }} \right| \\&= \underset{\varsigma \in [0,1]}{\text {max}} ~ r\left( \varsigma {\text {diag}}(v_\infty ) {\tilde{W}}^* \right) , \end{aligned}$$

where the last equality follows from the definition the numerical radius. Hence, the second addend in (70) is upper-bounded by

72 $$\begin{aligned} \underset{\varsigma \in [0,1], \upsilon \in {\mathcal {S}}}{\text {max}} ~ \frac{1}{\Vert {\tilde{\upsilon }} \Vert ^2_2} \left| {\tilde{\upsilon }}^T \varsigma {\text {diag}}(v_\infty ) {\tilde{W}}^* {\tilde{\upsilon }} \right|&\le r\left( \varsigma ^{(1)}_{\text {opt}} {\text {diag}}(v_\infty ) {\tilde{W}}^* \right) \end{aligned}$$

for some $\varsigma ^{(1)}_{\text {opt}} \in [0,1]$. Similarly, we obtain an upper bound on the third addend in (70) as

73 $$\begin{aligned} \underset{\varsigma \in [0,1], \upsilon \in {\mathcal {S}}}{\text {max}} ~\frac{1}{\Vert {\tilde{\upsilon }} \Vert ^2_2} \left| {\tilde{\upsilon }}^T {\text {diag}}(u - \varsigma v_\infty ) \varDelta {\tilde{W}} {\tilde{\upsilon }} \right| \le r\left( {\text {diag}}(u - \varsigma ^{(2)}_{\text {opt}} v_\infty ) \varDelta {\tilde{W}} \right) \end{aligned}$$

for some $\varsigma ^{(2)}_{\text {opt}} \in [0,1]$. With (71), (72) and (73), we obtain from (70) that

74 $$\begin{aligned} \varTheta _{\text {max}}( B, S )&\le \varTheta _{\text {max}} (B^*, S^* ) + r\left( \varsigma ^{(1)}_{\text {opt}} {\text {diag}}(v_\infty ) {\tilde{W}}^* \right) \nonumber \\&\quad + r\left( {\text {diag}}(u - \varsigma ^{(2)}_{\text {opt}} v_\infty ) \varDelta {\tilde{W}} \right) . \end{aligned}$$

The numerical radius *r*(*M*) is a vector^8^ norm (Horn and Johnson 1990) on the space of $N\times N$ matrices *M*. Thus, the numerical radius *r*(*M*) converges to zero if the matrix *M* converges to zero. Since $v_\infty \rightarrow 0$ and $\varDelta {\tilde{W}} \rightarrow 0$ as $(B, S) \rightarrow (B^*, S^*)$ and $\varsigma ^{(1)}_{\text {opt}}, \varsigma ^{(2)}_{\text {opt}}$ are bounded, the last two addends in (74) converge to zero as $(B, S) \rightarrow (B^*, S^*)$. Hence, for every scalar $\omega >0$ there is a $\vartheta (\omega )$ such that $\Vert B - B^* \Vert _2 < \vartheta (\omega )$ and $\Vert S - S^* \Vert _2 < \vartheta (\omega )$ implies that

75 $$\begin{aligned} \varTheta _{\text {max}}( B, S )\le \varTheta _{\text {max}}( B^*, S^* ) + \omega . \end{aligned}$$

We choose the scalar $\omega = (1- \varTheta _{\text {max}}( B^*, S^* ))/2$, which is positive due to (68). Then, the right-hand side of (75) becomes

$$\begin{aligned} \varTheta _{\text {max}}( B^*, S^* ) + \omega&= \frac{1}{2} + \frac{1}{2} \varTheta _{\text {max}}( B^*, S^* ) \\&= 1 - \omega . \end{aligned}$$

Thus, we obtain from (75) that

76 $$\begin{aligned} \varTheta _{\text {max}}( B, S ) \le 1 - \omega \end{aligned}$$

holds for all (*B*, *S*) which are sufficiently close to the limit $(B^*, S^*)$.

By definition, the error vector $\xi (t)$ at any time $t\ge 0$ is orthogonal to the steady-state vector $v_\infty$. Since the steady-state $v_\infty$ is positive, the error vector $\xi (t)$ has at least one positive and one negative element, and, hence, it holds that $\xi (t) \in {\mathcal {S}}$. Thus, we obtain from the definition of the term $\varTheta _{\text {max}}(B, S)$ in (66) that

$$\begin{aligned} \varTheta ( c(t), \xi (t), B, S ) \le \varTheta _{\text {max}}(B, S). \end{aligned}$$

With (76), we obtain from (64) that

$$\begin{aligned} \xi ^T(t)\varLambda _2(t) \le - \omega \Vert S^{\frac{1}{2}} \xi (t) \Vert ^2_2. \end{aligned}$$

From the sub-multiplicativity of the matrix norm, we obtain

$$\begin{aligned} \xi ^T(t)\varLambda _2(t) \le - \omega \Vert S^{\frac{1}{2}} \Vert ^2_2 \Vert \xi (t) \Vert ^2_2, \end{aligned}$$

which completes the proof, since $\Vert S^{\frac{1}{2}} \Vert ^2_2 = \delta _{\mathrm{max}}$. $\square$

### Bound on the error vector $\xi (t)$

With Lemma 4 and Lemma 7, we upper-bound (57) by

$$\begin{aligned} \frac{1}{2} \frac{d \Vert \xi (t)\Vert ^2_2}{d t } \le \frac{1}{4} \delta _{\text {max}}\left( \gamma ( R_0 - 1 ) + (R_0 + 1) \Vert \eta \Vert _2 \right) \Vert \xi (t)\Vert _2 - \omega \delta _{\text {max}} \Vert \xi (t) \Vert ^2_2. \end{aligned}$$

From

$$\begin{aligned} \frac{d \Vert \xi (t)\Vert _2}{d t }= \frac{1}{2 \Vert \xi (t)\Vert _2}\frac{d \Vert \xi (t)\Vert ^2_2}{d t }, \end{aligned}$$

it follows that

$$\begin{aligned} \frac{d \Vert \xi (t)\Vert _2}{d t } \le \frac{1}{4} \delta _{\text {max}}\left( \gamma ( R_0 - 1 ) + (R_0 + 1) \Vert \eta \Vert _2 \right) - \omega \delta _{\text {max}} \Vert \xi (t) \Vert _2. \end{aligned}$$

We denote

77 $$\begin{aligned} \varphi \left( B, S \right) = \frac{1}{4} \left( \gamma ( R_0 - 1 ) + (R_0 + 1) \Vert \eta \Vert _2 \right) , \end{aligned}$$

and we obtain that

78 $$\begin{aligned} \frac{d \Vert \xi (t)\Vert _2}{d t } \le \varphi \left( B, S \right) \delta _{\text {max}} - \omega \delta _{\text {max}} \Vert \xi (t) \Vert _2. \end{aligned}$$

The upper bound (78) is a linear first-order ordinary differential inequality, which is solved by (Arfken and Weber 1999)

$$\begin{aligned} \Vert \xi (t)\Vert _2 \le e^{-\omega \delta _{\text {max}} t} \left( \Vert \xi (0)\Vert _2 + \int ^{t}_0 \varphi \left( B, S \right) \delta _{\text {max}} e^{\omega \delta _{\text {max}} {\tilde{t}}} d {\tilde{t}}\right) , \end{aligned}$$

which simplifies to

$$\begin{aligned} \Vert \xi (t)\Vert _2 \le \left( \Vert \xi (0)\Vert _2 - \frac{\varphi \left( B, S \right) }{\omega }\right) e^{-\omega \delta _{\text {max}} t} + \frac{\varphi \left( B, S \right) }{\omega }. \end{aligned}$$

The triangle inequality yields that

79 $$\begin{aligned} \Vert \xi (t)\Vert _2 \le \Vert \xi (0)\Vert _2 e^{-\omega \delta _{\text {max}} t} + \frac{\varphi \left( B, S \right) }{\omega } \left( 1 +e^{-\omega \delta _{\text {max}} t} \right) . \end{aligned}$$

Furthermore, since $e^{-\omega \delta _{\text {max}} t} \le 1$ at every time $t\ge 0$, we obtain from (79) that

80 $$\begin{aligned} \Vert \xi (t)\Vert _2 \le \Vert \xi (0)\Vert _2 e^{-\omega \delta _{\text {max}} t} + 2 \frac{\varphi \left( B, S \right) }{\omega }. \end{aligned}$$

The maximum $\delta _{\text {max}}$ of the curing rates converges to some limit $\delta ^*_{\text {max}}$ when $\left( B, S\right) \rightarrow \left( B^*, S^*\right)$. Hence, for any $\epsilon >0$ it holds that $\delta ^*_{\text {max}} - \epsilon < \delta _{\text {max}}$ when $\left( B, S\right)$ approaches $\left( B^*, S^*\right)$. For some $\epsilon \in (0, \delta ^*_{\text {max}})$, we set the constant

$$\begin{aligned} \sigma _1 = \omega (\delta ^*_{\text {max}} - \epsilon ). \end{aligned}$$

Then, it holds that $\sigma _1 < \omega \delta _{\text {max}}$ when $\left( B, S\right)$ approaches $\left( B^*, S^*\right)$, and we obtain from (80) that

81 $$\begin{aligned} \Vert \xi (t)\Vert _2 \le \Vert \xi (0)\Vert _2 e^{-\sigma _1 t} + 2 \frac{\varphi \left( B, S \right) }{\omega }. \end{aligned}$$

Theorem 1 states that $\gamma = {\mathcal {O}}(R_0 - 1)$ and $\Vert \eta \Vert _2 = {\mathcal {O}}\left( (R_0 - 1)^2\right)$ when $\left( B, S\right)$ approaches $\left( B^*, S^*\right)$. Thus, it follows from the definition of the term $\varphi \left( B, S \right)$ in (77) that $\varphi \left( B, S \right) = {\mathcal {O}}\left( (R_0-1)^2\right)$. Hence, there is a constant $\sigma _2 >0$ such that (81) yields

$$\begin{aligned} \Vert \xi (t)\Vert _2 \le \Vert \xi (0)\Vert _2 e^{-\sigma _1 t} + \sigma _2 \left( R_0 - 1\right) ^2 \end{aligned}$$

when $$\left( B, S\right) $$ approaches $$\left( B^*, S^*\right) $$.

## Proof of Theorem 3

By projecting the differential equation (54) onto the steady-state vector $v_\infty$, we obtain that

$$\begin{aligned} \frac{d c(t)}{d t } v^T_\infty v_\infty = v^T_\infty \varLambda _1(t) + v^T_\infty \varLambda _2(t), \end{aligned}$$

since $v^T_\infty \xi (t) = 0$ by definition of the error term $\xi (t)$. We divide by $\Vert v_\infty \Vert ^2_2$ and obtain with (55) that

82 $$\begin{aligned} \frac{d c(t)}{d t } = \left( c(t) - c^2(t)\right) \frac{v^T_\infty \left( B - S \right) v_\infty }{\Vert v_\infty \Vert ^2_2} + \frac{v^T_\infty \varLambda _2(t)}{\Vert v_\infty \Vert ^2_2}. \end{aligned}$$

The first addend in the differential equation (82) can be expressed in a simpler manner when $\left( B, S\right)$ approaches $\left( B^*, S^* \right)$:

### Lemma 8

Under Assumptions 1 and 3, it holds

83 $$\begin{aligned} \frac{v^T_\infty \left( B - S \right) v_\infty }{v^T_\infty v_\infty } = (R_0 - 1) x_1^T S x_1 + \zeta , \end{aligned}$$

where $\zeta = {\mathcal {O}}\left( (R_0 - 1)^2\right)$ when $\left( B, S\right)$ approaches $\left( B^*, S^* \right)$.

### Proof

With Theorem 1 and the definition of the matrix *W* in (5), the numerator of the left-hand side of (83) becomes

$$\begin{aligned} v^T_\infty \left( B - S \right) v_\infty&= (\gamma x_1 + \eta )^T\left( S (W - I)\gamma x_1 + (B - S)\eta \right) \\&= (\gamma x_1 + \eta )^T (\gamma (R_0 - 1) S x_1 + (B - S) \eta ), \end{aligned}$$

where the last equality follows from $Wx_1 = R_0 x_1$. Thus, it holds that

84 $$\begin{aligned} v^T_\infty \left( B - S \right) v_\infty&= \gamma ^2 (R_0 - 1) x^T_1 S x_1 + \gamma x^T_1 (B - S) \eta \nonumber \\&\quad + \gamma (R_0 - 1) \eta ^T S x_1 + \eta ^T (B - S) \eta . \end{aligned}$$

Under Assumption 3, both matrices *B* and *S* are symmetric, which implies that

$$\begin{aligned} \left( x^T_1 (B - S)\right) ^T&= (B - S) x_1 \\&= S(R_0 - 1) x_1. \end{aligned}$$

Hence, we obtain from (84) that

$$\begin{aligned} v^T_\infty \left( B - S \right) v_\infty&= \gamma ^2 (R_0 - 1) x^T_1 S x_1 + \gamma (R_0 - 1) x^T_1 S \eta \nonumber \\&\quad + \gamma (R_0 - 1) \eta ^T S x_1 + \eta ^T (B - S) \eta . \end{aligned}$$

Since $\gamma = {\mathcal {O}}(R_0 - 1)$ and $\Vert \eta \Vert _2 = {\mathcal {O}}\left( (R_0 - 1)^2 \right)$, we finally rewrite the numerator of the left-hand side of (83) as

85 $$\begin{aligned} v^T_\infty \left( B - S \right) v_\infty =&\gamma ^2 (R_0 - 1) x^T_1 S x_1 + {\mathcal {O}}\left( (R_0 - 1)^4 \right) . \end{aligned}$$

With Theorem 1, the denominator of the left-hand side of (83) equals

86 $$\begin{aligned} v^T_\infty v_\infty&= \gamma ^2 + 2 \gamma \eta ^T x_1 + \Vert \eta \Vert ^2_2 \nonumber \\&= \gamma ^2 + {\mathcal {O}}\left( (R_0 - 1)^3 \right) . \end{aligned}$$

Combining the approximate expressions for the numerator (85) and the denominator (86) completes the proof. $\square$

We define the *viral slope* *w* as

87 $$\begin{aligned} w = (R_0 - 1) x_1^T S x_1 \end{aligned}$$

and the function *n*(*t*) as

88 $$\begin{aligned} n(t) = \left( c(t) - c^2(t)\right) \zeta + \frac{v^T_\infty \varLambda _2(t)}{\Vert v_\infty \Vert ^2_2}. \end{aligned}$$

Then, we obtain from (82) that

89 $$\begin{aligned} \frac{d c(t)}{d t } = \left( c(t) - c^2(t)\right) w + n(t). \end{aligned}$$

The function *n*(*t*) is complicated and depends on the error vector $\xi (t)$. Hence, we cannot solve the differential equation (89) for the function *c*(*t*) without knowing the solution for the error vector $\xi (t)$. However, as $(B, S) \rightarrow (B^*, S^*)$, the function *n*(*t*) converges to zero uniformly in time *t* as stated by the bound in Lemma 9.

### Lemma 9

Under Assumptions 1 to 3, it holds at every time $t \ge 0$ that

$$\begin{aligned} \left| n(t)\right| \le \sigma _1 \Vert \xi (0) \Vert _2 e^{-\sigma _2 t} + \sigma _3 (R_0 - 1)^2 \end{aligned}$$

for some constants $\sigma _1, \sigma _2, \sigma _3 >0$ when (*B*, *S*) approaches $(B^*, S^*)$.

### Proof

Regarding the first addend in the definition of the function *n*(*t*) in (88), Lemma 1 implies that $0 \le c(t) - c^2(t) \le 1/4$ at every time *t*. Hence, Lemma 8 yields that there is a constant ${\tilde{\sigma }}_0$ such that

$$\begin{aligned} \left| \left( c(t) - c^2(t)\right) \zeta \right| \le {\tilde{\sigma }}_0 (R_0 - 1)^2 \end{aligned}$$

at every time *t* when (*B*, *S*) approaches $(B^*, S^*)$. Regarding the second addend of the function *n*(*t*) defined in (88), it follows from the definition of the function $\varLambda _2(t)$ in (56) that

$$\begin{aligned} \frac{v^T_\infty \varLambda _2(t)}{\Vert v_\infty \Vert ^2_2}= \frac{1}{\Vert v_\infty \Vert ^2_2} v^T_\infty \left( B - S - c(t){\text {diag}}(B v_\infty ) - {\text {diag}}(v(t))B \right) \xi (t), \end{aligned}$$

since $v(t)=c(t)v_\infty + \xi (t)$. Thus, it holds that

$$\begin{aligned} \frac{v^T_\infty \varLambda _2(t)}{\Vert v_\infty \Vert ^2_2}&= \frac{1}{\Vert v_\infty \Vert ^2_2} v^T_\infty \left( - S + {\text {diag}}(u - v(t))B - c(t){\text {diag}}(B v_\infty ) \right) \xi (t). \end{aligned}$$

With the definition of the matrix ${\tilde{W}}$ in (32), we obtain that

$$\begin{aligned} \frac{v^T_\infty \varLambda _2(t)}{\Vert v_\infty \Vert ^2_2}= & {} \frac{1}{\Vert v_\infty \Vert ^2_2} v^T_\infty S^{\frac{1}{2}} \bigg ( -I + {\text {diag}}(u - v(t)){\tilde{W}} \\&- c(t)S^{-\frac{1}{2}}{\text {diag}}(B v_\infty ) S^{-\frac{1}{2}}\bigg )S^{\frac{1}{2}} \xi (t). \end{aligned}$$

The Cauchy–Schwarz inequality yields an upper bound as

$$\begin{aligned} \left| \frac{v^T_\infty \varLambda _2(t)}{\Vert v_\infty \Vert ^2_2}\right|&\le \frac{1}{\Vert v_\infty \Vert ^2_2} \left\Vert S^{\frac{1}{2}} \xi (t) \right\Vert _2\cdot \\&\quad \cdot \left\Vert \left( -I + {\text {diag}}(u - v(t)){\tilde{W}} - c(t)S^{-\frac{1}{2}}{\text {diag}}(B v_\infty ) S^{-\frac{1}{2}}\right) S^{\frac{1}{2}} v_\infty \right\Vert _2 \end{aligned}$$

With $\left\Vert S^{\frac{1}{2}} \xi (t) \right\Vert _2 \le \sqrt{\delta _{\text {max}}}\left\Vert \xi (t) \right\Vert _2$ and the triangle inequality, we obtain

90 $$\begin{aligned} \left| \frac{v^T_\infty \varLambda _2(t)}{\Vert v_\infty \Vert ^2_2}\right|&\le \sqrt{\delta _{\text {max}}} \frac{\left\Vert \xi (t) \right\Vert _2}{\Vert v_\infty \Vert ^2_2} \left\Vert \left( {\tilde{W}} - I \right) S^{\frac{1}{2}} v_\infty \right\Vert _2\nonumber \\&\quad +\sqrt{\delta _{\text {max}}} \frac{\left\Vert \xi (t) \right\Vert _2}{\Vert v_\infty \Vert ^2_2} \left\Vert {\text {diag}}(v(t)){\tilde{W}} \right\Vert _2 \left\Vert S^{\frac{1}{2}} v_\infty \right\Vert _2\nonumber \\&\quad +\sqrt{\delta _{\text {max}}} \frac{\left\Vert \xi (t) \right\Vert _2}{\Vert v_\infty \Vert ^2_2} \left\Vert c(t)S^{-\frac{1}{2}}{\text {diag}}(B v_\infty ) S^{-\frac{1}{2}} \right\Vert _2 \left\Vert S^{\frac{1}{2}} v_\infty \right\Vert _2. \end{aligned}$$

In the following, we consider the three addends in (90) separately. Regarding the first addend, we obtain with the definition of the matrix ${\tilde{W}}$ in (32) that

$$\begin{aligned} \left( {\tilde{W}} - I \right) S^{\frac{1}{2}} v_\infty&= S^{\frac{1}{2}} \left( W - I \right) v_\infty \\&= \gamma (R_0 - 1)S^{\frac{1}{2}} x_1 + S^{\frac{1}{2}} \left( W - I \right) \eta , \end{aligned}$$

where the last equality follows from Theorem 1. Thus, the triangle inequality yields

$$\begin{aligned} \left\Vert \left( {\tilde{W}} - I \right) S^{\frac{1}{2}} v_\infty \right\Vert _2 \le \gamma (R_0 - 1)\left\Vert S^{\frac{1}{2}} x_1\right\Vert _2 + \left\Vert S^{\frac{1}{2}} \left( W - I \right) \eta \right\Vert _2. \end{aligned}$$

With the sub-multiplicativity of the matrix 2-norm, we obtain

$$\begin{aligned} \left\Vert \left( {\tilde{W}} - I \right) S^{\frac{1}{2}} v_\infty \right\Vert _2 \le \sqrt{\delta _{\text {max}}} \left( \gamma (R_0 - 1) + (R_0 + 1) \left\Vert \eta \right\Vert _2\right) , \end{aligned}$$

since $\left\Vert \left( W - I \right) \right\Vert _2 \le R_0 + 1$. Since $\gamma = {\mathcal {O}}(R_0 - 1)$ and $\left\Vert \eta \right\Vert _2 = {\mathcal {O}}((R_0 - 1)^2)$ when $(B, S)\rightarrow (B^*, S^*)$, there is a constant ${\tilde{\sigma }}_1$ such that

91 $$\begin{aligned} \left\Vert \left( {\tilde{W}} - I \right) S^{\frac{1}{2}} v_\infty \right\Vert _2 \le {\tilde{\sigma }}_1 (R_0 - 1)^2 \end{aligned}$$

when (*B*, *S*) approaches $(B^*, S^*)$. Regarding the second addend in (90), it holds that

$$\begin{aligned} \left\Vert {\text {diag}}(v(t)){\tilde{W}} \right\Vert _2&\le \left\Vert {\text {diag}}(v(t))\right\Vert _2 \left\Vert {\tilde{W}} \right\Vert _2 \\&= R_0 \underset{l= 1,\ldots , N}{{\text {max}}} v_{\infty , l}. \end{aligned}$$

Since $\left\Vert v_{\infty }\right\Vert _2 = {\mathcal {O}}(R_0 - 1)$ when $(B, S)\rightarrow (B^*, S^*)$, it follows that there is a constant ${\tilde{\sigma }}_2$ such that

92 $$\begin{aligned} \left\Vert {\text {diag}}(v(t)){\tilde{W}} \right\Vert _2 \left\Vert S^{\frac{1}{2}} v_\infty \right\Vert _2&\le {\tilde{\sigma }}_2 (R_0 - 1)^2 \end{aligned}$$

when (*B*, *S*) approaches $(B^*, S^*)$. Regarding the third addend in (90), it holds per definition of the matrix 2-norm that

$$\begin{aligned} \left\Vert c(t)S^{-\frac{1}{2}}{\text {diag}}(B v_\infty ) S^{-\frac{1}{2}} \right\Vert _2&= c(t) \underset{l = 1,\ldots , N}{{\text {max}}} ~ \sum ^N_{j = 1} \frac{\beta _{jl}}{\delta _l} v_{\infty , j}\\&\le \underset{l = 1,\ldots , N}{{\text {max}}} ~ \left( W v_\infty \right) _l, \end{aligned}$$

where the last inequality follows from $c(t)\le 1$, as stated by Lemma 1, and the definition of the effective infection rate matrix *W* in (5). Hence, we obtain the upper-bound

93 $$\begin{aligned} \left\Vert c(t)S^{-\frac{1}{2}}{\text {diag}}(B v_\infty ) S^{-\frac{1}{2}} \right\Vert _2\left\Vert S^{\frac{1}{2}} v_\infty \right\Vert _2&\le {\tilde{\sigma }}_3 (R_0 - 1)^2 \end{aligned}$$

for some constant ${\tilde{\sigma }}_3$ when (*B*, *S*) approaches $(B^*, S^*)$. We apply the three upper bounds (91), (92) and (93) to (90) and obtain that

$$\begin{aligned} \left| \frac{v^T_\infty \varLambda _2(t)}{\Vert v_\infty \Vert ^2_2}\right| \le&\sqrt{\delta _{\text {max}}} \left( {\tilde{\sigma }}_1 + {\tilde{\sigma }}_2 + {\tilde{\sigma }}_3 \right) \frac{(R_0 - 1)^2}{\Vert v_\infty \Vert ^2_2} \left\Vert \xi (t) \right\Vert _2 \end{aligned}$$

when (*B*, *S*) approaches $(B^*, S^*)$. Since $\left\Vert v_{\infty }\right\Vert ^2_2 = {\mathcal {O}}((R_0 - 1)^2)$ when $(B, S)\rightarrow (B^*, S^*)$, there is a constant ${\tilde{\sigma }}_4$ such that, as (*B*, *S*) approaches $(B^*, S^*)$, it holds

$$\begin{aligned} \left| \frac{v^T_\infty \varLambda _2(t)}{\Vert v_\infty \Vert ^2_2} \right| \le&{\tilde{\sigma }}_4 \left\Vert \xi (t) \right\Vert _2 \end{aligned}$$

at every time *t*. Thus, we have obtained an upper bound, which is proportional to the norm of the error vector $\xi (t)$. Finally, we apply Theorem 2 to bound the norm $\left\Vert \xi (t) \right\Vert _2$, which completes the proof. $\square$

Lemma 9 suggests that, since $n(t)\rightarrow 0$ when $\left( B, S\right) \rightarrow \left( B^*, S^*\right)$, the differential equation (89) for the function *c*(*t*) is approximated by the logistic differential equation

94 $$\begin{aligned} \frac{d c(t)}{d t } \approx \left( c(t) - c^2(t)\right) w. \end{aligned}$$

To make the statement (94) precise, we define the function $c_b(t, x)$, for any scalar *x* with $|x|<w$, as

95 $$\begin{aligned} c_b(t, x) = \frac{1}{2} + \frac{1}{2}\sqrt{ 1 + \frac{x}{w}} \tanh \left( \frac{ \sqrt{w(w + x)}}{2} t + \varUpsilon (x)\right) , \end{aligned}$$

where the constant $\varUpsilon (x)$ is set such that $c_b(0, x) = c(0)$, i.e.,

$$\begin{aligned} \varUpsilon (x) = {\mathrm{artanh}} \left( \frac{2w}{\sqrt{w(w+x)}} \left( c(0) -\frac{1}{2} \right) \right) . \end{aligned}$$

Lemma 10 states an upper and a lower bound on the function *c*(*t*).

### Lemma 10

Suppose that Assumptions 1 to 3 hold and that

96 $$\begin{aligned} \Vert \xi (0) \Vert _2 \le \sigma _1 (R_0 - 1)^p \end{aligned}$$

for some constants $\sigma _1>0$ and $p>1$ when $\left( B, S\right)$ approaches $\left( B^*, S^*\right)$. Then, the function *c*(*t*) is bounded by

$$\begin{aligned} c_b(t, -\kappa ) \le c(t) \le c_b(t, \kappa ) \quad \forall ~ t\ge 0, \end{aligned}$$

where the scalar $\kappa$ equals $\kappa = \sigma _2 (R_0 - 1)^s$ with $s = {\mathrm{min}}\{p, 2\}$ and some constant $\sigma _2 >0$ as $\left( B, S\right)$ approaches $\left( B^*, S^*\right)$.

### Proof

With (96), Lemma 9 implies that it holds

$$\begin{aligned} \left| n(t)\right| \le {\tilde{\sigma }}_1 (R_0 - 1)^p e^{-{\tilde{\sigma }}_2 t} + {\tilde{\sigma }}_3 (R_0 - 1)^2 \end{aligned}$$

for some constants ${\tilde{\sigma }}_1, {\tilde{\sigma }}_2, {\tilde{\sigma }}_3>0$. Since $e^{-{\tilde{\sigma }}_2 t}\le 1$, we obtain that $\left| n(t)\right| \le \kappa$ at every time *t*, where we define the scalar

$$\begin{aligned} \kappa = {\tilde{\sigma }}_4 (R_0 - 1)^s \end{aligned}$$

with the constants $s = {\mathrm{min}}\{p, 2\}$ and ${\tilde{\sigma }}_4 = {\tilde{\sigma }}_1 + {\tilde{\sigma }}_3$. With $\left| n(t)\right| \le \kappa$, we obtain from the differential equation (89) for the function *c*(*t*) that

97 $$\begin{aligned} \left( c(t) - c^2(t)\right) w - \kappa \le \frac{d c(t)}{d t } \le \left( c(t) - c^2(t)\right) w + \kappa \quad \forall ~ t\ge 0. \end{aligned}$$

The upper and lower bound (97) give rise to a Riccati differential equation, which can be solved exactly, and we obtain that the function *c*(*t*) is bounded by

$$\begin{aligned} c(t) \ge \frac{1}{2} + \frac{1}{2}\sqrt{ 1 - \frac{\kappa }{w}} \tanh \left( \frac{\sqrt{w(w - \kappa )}}{2}t + \varUpsilon (-\kappa )\right) \end{aligned}$$

and

$$\begin{aligned} c(t) \le \frac{1}{2} + \frac{1}{2}\sqrt{ 1 + \frac{\kappa }{w}} \tanh \left( \frac{\sqrt{w(w + \kappa )} }{2}t + \varUpsilon (\kappa )\right) . \end{aligned}$$

at every time $t\ge 0$. $\square$

When $\left( B, S\right)$ approaches $\left( B^*, S^*\right)$, Theorem 2 states that the error term $\xi (t)$ is negligible and, furthermore, Lemma 10 states that the function *c*(*t*) converges to $c_b(t, 0)$. Thus, based on the ansatz (15), we approximate the viral state *v*(*t*) by

$$\begin{aligned} v_{\text {apx}}(t)&= c_b(t, 0) v_\infty . \end{aligned}$$

With the definition of the function $c_b(t, x)$ in (95), it holds that

$$\begin{aligned} v_{\text {apx}}(t)&= \frac{1}{2} \left( 1 + \tanh \left( \frac{ w }{2} + \varUpsilon (0)\right) \right) v_\infty . \end{aligned}$$

Then, it follows from the ansatz (15) that the difference of the exact viral state *v*(*t*) to the approximation $v_{\text {apx}}(t)$ equals

98 $$\begin{aligned} \Vert v(t) - v_{\text {apx}}(t) \Vert _2 = \left| c(t) - c_b(t, 0)\right| \Vert v_\infty \Vert _2 + \Vert \xi (t) \Vert _2. \end{aligned}$$

The norm $\Vert \xi (t) \Vert _2$ of the error term $\xi (t)$ is bounded by Theorem 2. Thus, it remains to bound the first addend of (98). With Lemma 10, the difference of the function *c*(*t*) to $c_b(t, 0)$ is bounded by

99 $$\begin{aligned} \left| c(t) - c_b(t, 0) \right| \le c_b(t, \kappa ) - c_b(t, -\kappa ). \end{aligned}$$

Furthermore, the scalar $\kappa$ converges to zero when $\left( B, S\right)$ approaches $\left( B^*, S^*\right)$. Hence, if we show that, as the scalar $\kappa$ converges to zero, the upper bound $c_b(t, \kappa )$ converges to the lower bound $c_b(t, -\kappa )$ then (99) implies that the function *c*(*t*) converges to $c_b(t, 0)$. Furthermore, we must show that the upper bound $c_b(t, \kappa )$ converges to the lower bound $c_b(t, -\kappa )$ *uniformly in time* *t*, since the upper bound on the approximation error $\Vert v(t) - v_{\mathrm{apx}}(t)\Vert _2$ in Theorem 3 does not depend on time *t*. From the definition of the function $c_b(t, x)$ in (95) we obtain that

100 $$\begin{aligned} \left| c(t) - c_b(t, 0) \right|&\le \frac{1}{2}\sqrt{ 1 + \frac{\kappa }{w}} g(t, \kappa )- \frac{1}{2}\sqrt{ 1 - \frac{\kappa }{w}} g(t, -\kappa ), \end{aligned}$$

where we denote

101 $$\begin{aligned} g(t, \kappa ) = \tanh \left( \frac{ \sqrt{w\left( w + \kappa \right) }}{2}t + \varUpsilon \left( \kappa \right) \right) . \end{aligned}$$

Lemma 10 states that $\kappa = {\mathcal {O}}((R_0 - 1)^s)$ for some $s>1$ when $\left( B, S\right)$ approaches $\left( B^*, S^*\right)$. Furthermore, Lemma 8 states that $w = {\mathcal {O}}(R_0 - 1)$. Hence, it holds that $\kappa /w = {\mathcal {O}}((R_0 - 1)^{s -1})$ when $\left( B, S\right)$ approaches $\left( B^*, S^*\right)$. For small *x*, the series expansion of the square root yields that

$$\begin{aligned} \frac{1}{2}\sqrt{ 1 + x} = \frac{1}{2} + \frac{1}{4} x + {\mathcal {O}}(x^2). \end{aligned}$$

Thus, for small values of $\kappa /w$, we obtain from (100) that

$$\begin{aligned} \left| c(t) - c_b(t, 0) \right|&\le \frac{1}{2} \left( g(t, \kappa ) - g(t, -\kappa ) \right) + \frac{1}{4 w} \kappa \left( g(t, \kappa ) + g(t, -\kappa ) \right) \\&\quad +\left( g(t, \kappa ) - g(t, -\kappa ) \right) \cdot {\mathcal {O}}\left( \frac{\kappa ^2}{w^2}\right) . \end{aligned}$$

Since the magnitude of the hyperbolic tangent is bounded by 1, it follows from the definition of the function $g(t, \kappa )$ in (101) that

$$\begin{aligned} \left| g(t, \kappa ) - g(t, -\kappa ) \right| \le \left| g(t, \kappa )\right| + \left| g(t, -\kappa ) \right| \le 2, \end{aligned}$$

which yields that

102 $$\begin{aligned} \left| c(t) - c_b(t, 0) \right| \le&\frac{1}{2} \left( g(t, \kappa ) - g(t, -\kappa ) \right) + \frac{1}{2 w} \kappa + {\mathcal {O}}\left( (R_0 - 1)^{2(s - 1)}\right) , \end{aligned}$$

since $\kappa /w = {\mathcal {O}}((R_0 - 1)^{s -1})$. The last two addends of (102) are independent of time *t*. Thus, it remains to show that first addend, i.e., the difference $(g(t, \kappa ) - g(t, -\kappa ))$, converges to zero *uniformly in time* *t* as $\kappa \rightarrow 0$.

### Lemma 11

Under Assumptions 1 to 3, there is some constant $\sigma _1>0$ such that

$$\begin{aligned} \left| g(t, \kappa ) - g(t, -\kappa )\right| \le 2 \sigma _1 \kappa \end{aligned}$$

at every time $t\ge 0$ when the scalar $\kappa$ approaches zero from above.

### Proof

The mean value theorem gives that

$$\begin{aligned} g(t, \kappa ) = g(t, 0)+ \left. \partial _\kappa g(t, \kappa )\right| _{\kappa =z(t)} \kappa \end{aligned}$$

for some $z(t)\in (0, \kappa )$. Thus, it holds that

$$\begin{aligned} g(t, \kappa ) - g(t, -\kappa ) = \left. \partial _\kappa g(t, \kappa )\right| _{\kappa =z_1(t)} \kappa + \left. \partial _\kappa g(t, \kappa )\right| _{\kappa =z_2(t)} \kappa \end{aligned}$$

for some $z_1(t)\in (0, \kappa )$ and $z_2(t)\in (- \kappa , 0)$, which yields that

103 $$\begin{aligned} \left| g(t, \kappa ) - g(t, -\kappa )\right| = \left| \left. \partial _\kappa g(t, \kappa )\right| _{\kappa =z_1(t)}\right| \kappa + \left| \left. \partial _\kappa g(t, \kappa )\right| _{\kappa =z_2(t)}\right| \kappa . \end{aligned}$$

To express the derivative of the function $g(t, \kappa )$, we write the function *g*(*t*, *x*) as

$$\begin{aligned} g(t, \kappa ) = \tanh (h(t, \kappa )), \end{aligned}$$

where we define the function $h(t, \kappa )$ as

$$\begin{aligned} h(t, \kappa ) = \frac{ \sqrt{w\left( w + \kappa \right) }}{2}t + \varUpsilon \left( \kappa \right) . \end{aligned}$$

Then, the derivative of the function $g(t, \kappa )$ with respect to the scalar $\kappa$ is given by

$$\begin{aligned} \partial _\kappa g(t, \kappa ) = \frac{4}{\left( e^{-h(t, \kappa )} + e^{h(t, \kappa )}\right) ^2} \partial _\kappa h(t, \kappa ), \end{aligned}$$

which is upper-bounded by

104 $$\begin{aligned} \left| \partial _\kappa g(t, \kappa )\right| \le 4 e^{-2 h(t, \kappa )} \left| \partial _\kappa h(t, \kappa ) \right| . \end{aligned}$$

With the derivative of the function $h(t, \kappa )$, i.e.

$$\begin{aligned} \partial _\kappa h(t, \kappa ) = \frac{w}{4 \sqrt{ w\left( w + \kappa \right) }} t + \partial _\kappa \varUpsilon \left( \kappa \right) , \end{aligned}$$

we obtain from (104) that

$$\begin{aligned} \left| \partial _\kappa g(t, \kappa )\right| \le 4 e^{-\sqrt{w\left( w + \kappa \right) }t -2 \varUpsilon \left( \kappa \right) }\left| \frac{w}{4 \sqrt{ w\left( w + \kappa \right) }} t + \partial _\kappa \varUpsilon \left( \kappa \right) \right| . \end{aligned}$$

The right-hand side of (104) is finite at every time $t\ge 0$. Furthermore, for every scalar $\kappa$, the right-hand side of (104) converges to zero when $t\rightarrow \infty$. Hence, we can upper-bound the derivative $\left| \partial _\kappa g(t, \kappa )\right|$ by some constant $\sigma _1>0$ for every time *t*. Thus, we obtain from (103) that

$$\begin{aligned} \left| g(t, \kappa ) - g(t, -\kappa )\right| = 2\sigma _1 \kappa \quad \forall ~ t\ge 0. \end{aligned}$$

$$\square $$

With Lemma 11, we obtain from (102) that there is a constant $\sigma _1 >0$ such that

$$\begin{aligned} \left| c(t) - c_b(t, 0) \right| \le&\sigma _1 \kappa + \frac{1}{2} \frac{\kappa }{w} + {\mathcal {O}}\left( (R_0 - 1)^{2(s - 1)}\right) \quad \forall ~t\ge 0. \end{aligned}$$

Since $\kappa = {\mathcal {O}}((R_0 - 1)^s)$ and $w = {\mathcal {O}}(R_0 - 1)$ when $\left( B, S\right)$ approaches $\left( B^*, S^*\right)$, we obtain that there exists some constant $\sigma _2 >0$ such that

$$\begin{aligned} \left| c(t) - c_b(t, 0) \right| \le&\sigma _2 (R_0 - 1)^{s-1}. \end{aligned}$$

Thus, it follows from (98) that

$$\begin{aligned} \Vert v(t) - v_{\text {apx}}(t) \Vert _2 \le \sigma _2 (R_0 - 1)^{s-1} \Vert v_\infty \Vert _2 + \Vert \xi (t) \Vert _2, \quad \forall t\ge 0. \end{aligned}$$

Hence, we obtain an upper bound as

$$\begin{aligned} \frac{\Vert v(t) - v_{\mathrm{apx}}(t)\Vert _2}{ \Vert v_\infty \Vert _2} \le \sigma _2 (R_0 - 1)^{s-1} + \frac{\Vert \xi (t) \Vert _2}{ \Vert v_\infty \Vert _2}. \end{aligned}$$

Then, the upper bound on the error vector $\xi (t)$ in Theorem 2 implies that there are constants $\sigma _3, \sigma _4$ such that

$$\begin{aligned} \frac{\Vert v(t) - v_{\mathrm{apx}}(t)\Vert _2}{ \Vert v_\infty \Vert _2} \le&\sigma _2 (R_0 - 1)^{s-1} +\frac{ \Vert \xi (0) \Vert _2}{ \Vert v_\infty \Vert _2} e^{-\sigma _3 t}+\sigma _4 \frac{ (R_0 - 1)^2}{ \Vert v_\infty \Vert _2}. \end{aligned}$$

By assumption it holds that $\Vert \xi (0) \Vert _2 = {\mathcal {O}}\left( (R_0-1)^p\right)$ for some constant $p>1$, and it holds that $\Vert v_\infty \Vert _2 = {\mathcal {O}}(R_0-1)$ as stated by Theorem 1. Thus, we obtain that

$$\begin{aligned} \frac{\Vert v(t) - v_{\mathrm{apx}}(t)\Vert _2}{ \Vert v_\infty \Vert _2} \le&\sigma _2 (R_0 - 1)^{s-1} +\sigma _5 (R_0-1)^{p-1} +\sigma _6 (R_0 - 1) \end{aligned}$$

for some constants $\sigma _5, \sigma _6>0$, since $e^{-\sigma _3 t}\le 1$. By using the definition $s = {\text {min}}\{p, 2\}$ of the scalar *s*, we complete the proof.

## Proof of Corollary 1

By assumption, it holds that $v(0)=r_0 v_\infty$, which implies that $\xi (0)= 0$. Thus, we obtain from (22) that

$$\begin{aligned} \frac{\Vert v(t) - v_{\mathrm{apx}}(t)\Vert _2}{ \Vert v_\infty \Vert _2} \le \sigma _1 (R_0 - 1 ) \quad \forall t\ge 0 \end{aligned}$$

when $\left( B, S\right)$ approaches $\left( B^*, S^*\right)$. From the definition of the approximation $v_{{\text {apx}}}(t)$ in (20), we obtain that $v_{{\text {apx}}, i}(t_{01}) = r_1 v_{\infty , i}$ for every node *i* is equivalent to

$$\begin{aligned} \tanh \left( \frac{ w}{2} t_{01} + \varUpsilon (0)\right) = 2 r_1 - 1 \end{aligned}$$

With the definition of the term $\varUpsilon (0)$ in (19), it follows that

$$\begin{aligned} \frac{ w}{2} t_{01} = {\text {artanh}}( 2 r_1 - 1 ) - {\text {artanh}} \left( 2 \frac{ v^T_\infty v(0) }{ \Vert v_\infty \Vert ^2_2 } -1 \right) . \end{aligned}$$

From $v(0) = r_0 v_\infty$, we obtain that

$$\begin{aligned} t_{01} = \frac{2}{w} \left( {\text {artanh}}( 2 r_1 - 1 ) - {\text {artanh}} \left( 2 r_0 -1 \right) \right) . \end{aligned}$$

The inverse hyperbolic tangent equals

$$\begin{aligned} {\text {artanh}}(x) = \frac{1}{2}\left( \log (1+x) - \log (1-x)\right) , \end{aligned}$$

which completes the proof.

## Proof of Corollary 3

For NIMFA (4) with homogeneous spreading parameters $\beta , \delta$, the effective infection rate matrix reduces to $W = \frac{\beta }{\delta } A$. Hence, the basic reproduction number reproduction becomes

$$\begin{aligned} R_0 = \frac{\beta }{\delta } \rho (A) = \frac{\tau }{\tau _c}, \end{aligned}$$

where the last equation follows from the definition of the effective infection rate $\tau = \beta / \delta$ and the epidemic threshold $\tau _c = 1/\rho (A)$. Furthermore, it holds that $\delta _l = \delta$ for every node *l* and $\sum ^N_{l=1}(x_1)^2_l = 1$, since the principal eigenvector $x_1$ is of unit length. Thus, the definition of the approximation $v_{{\text {apx}}}(t)$ in (20) yields that

$$\begin{aligned} v_{\text {apx}}(t) = \frac{1}{2} \left( 1 + \tanh \left( \frac{ (\tau - \tau _c) \delta }{2\tau _c} t + \varUpsilon (0)\right) \right) v_\infty . \end{aligned}$$

## Proof of Theorem 4

We acknowledge the help of Karel Devriendt, who constructed an effective infection rate matrix of homogeneous NIMFA with a given principal eigenvector $x_1$. The idea of proving Theorem 4 is based on Corollary 2: When $R_0\downarrow 1$, the viral state dynamics of heterogeneous NIMFA (1) are determined by the four variables $x_1,w, \gamma , \varUpsilon (0)$. Thus, we aim to show that the corresponding four variables of the homogeneous NIMFA system (26), which we denote by $x_{1,{\text {hom}}},w_{\text {hom}}, \gamma _{\text {hom}}$ and $\varUpsilon _{\text {hom}}(0)$, are the same as the variables $x_1,w, \gamma , \varUpsilon (0)$ of heterogeneous NIMFA (1).

### Lemma 12

The homogeneous NIMFA system (26) and heterogeneous NIMFA (1) have the same principal eigenvector $x_{1, {{\mathrm{hom}}}}=x_1$, the variable $\gamma _{{\mathrm{hom}}}=\gamma$ and viral slope $w_{{\mathrm{hom}}}=w$.

### Proof

First, we consider the principal eigenvector $x_1$. The effective infection rate matrix of the homogeneous NIMFA system (26) equals

105 $$\begin{aligned} W_{\text {hom}}&= \frac{\beta _{\text {hom}}}{\delta _{\text {hom}}} u u^T +\frac{\beta _{\text {hom}}}{\delta _{\text {hom}}} \frac{1}{\underset{l=1,\ldots , N}{{\text {min}}} \left( x_1\right) _l } \sum ^N_{j=1} \left( x_1\right) _j I \nonumber \\&\quad -\frac{\beta _{\text {hom}}}{\delta _{\text {hom}}} \sum ^N_{j=1} \left( x_1\right) _j{\text {diag}}\left( \frac{1}{\left( x_1\right) _1},\ldots , \frac{1}{\left( x_1\right) _N}\right) . \end{aligned}$$

We show that the principal eigenvector $x_1$ of heterogeneous NIMFA (1) is also the principal eigenvector $x_{1, {\text {hom}}}$ of the matrix $W_{\text {hom}}$. Indeed,

$$\begin{aligned} W_{\text {hom}} x_1&= \frac{\beta _{\text {hom}}}{\delta _{\text {hom}}} \sum ^N_{j=1} \left( x_1\right) _j u+\frac{\beta _{\text {hom}}}{\delta _{\text {hom}}} \frac{1}{\underset{l=1,\ldots , N}{{\text {min}}} \left( x_1\right) _l } \sum ^N_{j=1} \left( x_1\right) _j x_1\\&\quad -\frac{\beta _{\text {hom}}}{\delta _{\text {hom}}} \sum ^N_{j=1} \left( x_1\right) _j u \\&= \frac{\beta _{\text {hom}}}{\delta _{\text {hom}}} \frac{1}{\underset{l=1,\ldots , N}{{\text {min}}} \left( x_1\right) _l } \sum ^N_{j=1} \left( x_1\right) _j x_1. \end{aligned}$$

Thus, $x_1$ is an eigenvector of the effective infection rate matrix $W_{\text {hom}}$ of the homogeneous NIMFA system (26). The corresponding eigenvalue equals

106 $$\begin{aligned} \lambda _{1, {\text {hom}}} = \frac{\beta _{\text {hom}}}{\delta _{\text {hom}}} \frac{1}{\underset{l=1,\ldots , N}{{\text {min}}} \left( x_1\right) _l } \sum ^N_{j=1} \left( x_1\right) _j . \end{aligned}$$

The effective infection rate matrix $W_{\text {hom}}$ is non-negative and irreducible, by definition (105). Thus, the Perron–Frobenius Theorem (Van Mieghem 2010) yields that the eigenvalue $\lambda _{1, {\text {hom}}}$ to the positive eigenvector $x_1$ equals the spectral radius $\rho \left( W_{\text {hom}}\right) =\lambda _{1, {\text {hom}}}$ and that $x_{1,{\text {hom}}}=x_1$. Second, we consider the variables $\gamma$, $\gamma _{\text {hom}}$ in Theorem 1. By definition (7) and since $x_1$ is a vector of length 1, it holds that

$$\begin{aligned} \gamma _{\text {hom}}&= \left( \lambda _{1, {\text {hom}}} - 1\right) \frac{1}{\sum ^N_{l=1}\left( x_1\right) ^3_l} \\&= \left( \frac{\beta _{\text {hom}}}{\delta _{\text {hom}}} \frac{1}{\underset{l=1,\ldots , N}{{\text {min}}} \left( x_1\right) _l }\sum ^N_{j=1}\left( x_1\right) _j -1\right) \frac{1}{\sum ^N_{p=1}\left( x_1\right) ^3_p}, \end{aligned}$$

where the last equality follows from (106). With (28), we obtain further that

$$\begin{aligned} \gamma _{\text {hom}}&= \left( \left( 1 + \gamma \sum ^N_{l=1} \left( x_1\right) ^3_l \right) -1\right) \frac{1}{\sum ^N_{p=1}\left( x_1\right) ^3_p} \\&= \gamma . \end{aligned}$$

Thus, the variable $\gamma _{\text {hom}}$ of the homogeneous NIMFA (26) equals the variable $\gamma$ of heterogeneous NIMFA (1). Third, we show that the viral slope $w_{\text {hom}}$ of the homogeneous NIMFA (26) equals the viral slope *w* of heterogeneous NIMFA (1). From the definition (87), the variable $w_{\text {hom}}$ of the homogeneous NIMFA system (26) follows as

$$\begin{aligned} w_{\text {hom}}= \left( \lambda _{1, {\text {hom}}} - 1 \right) \delta _{\text {hom}}. \end{aligned}$$

With (106), we obtain that

$$\begin{aligned} w_{\text {hom}}= \beta _{\text {hom}} \frac{1}{\underset{l=1,\ldots , N}{{\text {min}}} \left( x_1\right) _l } \sum ^N_{j=1} \left( x_1\right) _j - \delta _{\text {hom}}. \end{aligned}$$

Then, the definition of the infection rate $\beta _{\text {hom}}$ in (28) yields that

$$\begin{aligned} w_{\text {hom}}&= \delta _{\text {hom}} \left( 1 + \gamma \sum ^N_{l=1} \left( x_1\right) ^3_l \right) - \delta _{\text {hom}}\\&= \delta _{\text {hom}} \gamma \sum ^N_{l=1} \left( x_1\right) ^3_l, \end{aligned}$$

which simplifies with the definition of $\delta _{\text {hom}}$ in (27) to

$$\begin{aligned} w_{\text {hom}}&= \gamma \sum ^N_{l=1} \delta _l \left( x_1\right) ^3_l. \end{aligned}$$

Then, the definition of $\gamma$ in (7) yields that

$$\begin{aligned} w_{\text {hom}}&= \left( R_0 - 1 \right) \sum ^N_{l=1} \delta _l \left( x_1\right) ^2_l. \end{aligned}$$

Thus, the viral slope $w_{\text {hom}}$ of the homogeneous NIMFA system (26) equals the viral slope *w* of heterogeneous NIMFA (1), which completes the proof. $\square$

In contrast to the variables $x_1, \gamma , w$ in Lemma 12, the two variables $\varUpsilon _{\text {hom}}(0)$ and $\varUpsilon (0)$, given by definition (19), are not necessarily equal, since the steady states $v_\infty$ and $v_{\infty , {\text {hom}}}$ might be different. For the homogeneous NIMFA system (26) and heterogeneous NIMFA (1), we denote the viral state approximations of Corollary 2 by ${\tilde{v}}_{\text {apx}}(t)$ and ${\tilde{v}}_{\text {apx,hom}}(t)$, respectively. The difference of the viral state vectors *v*(*t*) and $v_{\text {hom}}(t)$ can be written as

$$\begin{aligned} v(t) - v_{\text {hom}}(t)&= {\tilde{v}}_{\text {apx}}(t) - {\tilde{v}}_{\text {apx,hom}}(t)+\left( v(t) - {\tilde{v}}_{\text {apx}}(t) \right) \\&\quad -\left( v_{\text {hom}}(t) - {\tilde{v}}_{\text {apx,hom}}(t) \right) . \end{aligned}$$

With the triangle inequality, we obtain that

107 $$\begin{aligned} \left\Vert v(t) - v_{\text {hom}}(t)\right\Vert _2&\le \left\Vert {\tilde{v}}_{\text {apx}}(t) - {\tilde{v}}_{\text {apx,hom}}(t) \right\Vert _2 +\left\Vert v(t) - {\tilde{v}}_{\text {apx}}(t) \right\Vert _2 \nonumber \\&\quad +\left\Vert v_{\text {hom}}(t) - {\tilde{v}}_{\text {apx,hom}}(t) \right\Vert _2 . \end{aligned}$$

Corollary 2 states that there is some constant $\sigma$, such that, at every time $t\ge 0$, it holds that

$$\begin{aligned} \Vert v(t) - {\tilde{v}}_{\text {apx}}(t)\Vert _2&\le \sigma \Vert v_\infty \Vert _2 (R_0 - 1)^{s-1}\\&={\mathcal {O}}\left( (R_0 - 1)^s \right) \end{aligned}$$

as $R_0\downarrow 1$, since $\Vert v_\infty \Vert _2 = {\mathcal {O}}\left( R_0 - 1 \right)$ by Theorem 1. Similarly, Corollary 2 implies that $\Vert v_{\text {hom}}(t) - {\tilde{v}}_{\text {apx, hom}}(t)\Vert _2 = {\mathcal {O}}\left( (R_0 - 1)^s \right)$ as $R_0 \downarrow 1$. Thus, (107) yields that

108 $$\begin{aligned} \left\Vert v(t) - v_{\text {hom}}(t)\right\Vert _2 \le&\left\Vert v_{\text {apx}}(t) - v_{\text {apx,hom}}(t) \right\Vert _2 +{\mathcal {O}}\left( (R_0 - 1)^s \right) . \end{aligned}$$

In the following, we bound the first addend on the right side of (108). We insert the expression (23) for the approximations $v_{\text {apx}}(t)$ and $v_{\text {apx,hom}}(t)$ to obtain that

$$\begin{aligned} v_{\text {apx}}(t) - v_{\text {apx,hom}}(t)&= \left( 1 + \tanh \left( \frac{w}{2} t + \varUpsilon (0) \right) \right) \frac{\gamma }{2} x_1 \\&\quad - \left( 1 + \tanh \left( \frac{w_{\text {hom}}}{2} t + \varUpsilon _{\text {hom}}(0) \right) \right) \frac{\gamma _{\text {hom}}}{2} x_{1,{\text {hom}}}\\&= \left( \tanh \left( \frac{w}{2} t + \varUpsilon (0) \right) - \tanh \left( \frac{w}{2} t + \varUpsilon _{\text {hom}}(0) \right) \right) \frac{\gamma }{2} x_1, \end{aligned}$$

where the second equality follows from Lemma 12. From Abramowitz and Stegun (1965, 4.5.45), it follows that

$$\begin{aligned} v_{\text {apx}}(t) - v_{\text {apx,hom}}(t)&= {\text {sech}}\left( \frac{w}{2} t + \varUpsilon (0) \right) {\text {sech}}\left( \frac{w}{2} t + \varUpsilon _{\text {hom}}(0) \right) \\&\quad \cdot \sinh \left( \varUpsilon (0) - \varUpsilon _{\text {hom}}(0) \right) \frac{\gamma }{2} x_1. \end{aligned}$$

Since $0< {\text {sech}} (t)\le 1$ for every time *t* and the eigenvector $x_1$ has length 1, we obtain that

$$\begin{aligned} \left\Vert v_{\text {apx}}(t) - v_{\text {apx,hom}}(t) \right\Vert _2&\le \frac{\gamma }{2} \left| \sinh \left( \varUpsilon (0) - \varUpsilon _{\text {hom}}(0) \right) \right| . \end{aligned}$$

Thus, the difference of the viral states *v*(*t*) and $v_{\text {hom}}(t)$ in (108) is bounded by

109 $$\begin{aligned} \left\Vert v(t) - v_{\text {hom}}(t)\right\Vert _2&\le \frac{\gamma }{2} \left| \sinh \left( \varUpsilon (0) - \varUpsilon _{\text {hom}}(0) \right) \right| +{\mathcal {O}}\left( (R_0 - 1)^s \right) . \end{aligned}$$

To bound the hyperbolic sine on the right side of (109), we introduce:

### Lemma 13

Suppose that Assumptions 1 to 3 hold. Furthermore, assume that the initial viral states of the homogeneous NIMFA system (26) and heterogeneous NIMFA (1) are the same, i.e., $v(0)=v_{{\mathrm{hom}}}(0)$. Then, as $R_0\downarrow 1$, it holds that

$$\begin{aligned} \left| \sinh \left( \varUpsilon (0) - \varUpsilon _{{\mathrm{hom}}}(0) \right) \right| = {\mathcal {O}}\left( R_0 - 1 \right) . \end{aligned}$$

### Proof

The series expansion (Abramowitz and Stegun 1965, 4.5.62) of the hyperbolic sine yields that

110 $$\begin{aligned} \sinh \left( \varUpsilon (0) - \varUpsilon _{{\mathrm{hom}}}(0) \right) = \varUpsilon (0) - \varUpsilon _{{\mathrm{hom}}}(0) + {\mathcal {O}}\left( \left( \varUpsilon (0) - \varUpsilon _{{\mathrm{hom}}}(0) \right) ^3\right) . \end{aligned}$$

In the following, we consider the difference $\varUpsilon (0) - \varUpsilon _{{\mathrm{hom}}}(0)$. Since $v(0)=v_{\text {hom}}(0)$ by the assumption, it follows from the definition of the variable $\varUpsilon (0)$ in (19) that

111 $$\begin{aligned} \varUpsilon (0) - \varUpsilon _{\text {hom}}(0)&= {\text {artanh}}\left( 2 \frac{ v^T_\infty v(0) }{ \Vert v_\infty \Vert ^2_2 } -1 \right) -{\text {artanh}}\left( 2 \frac{ v^T_{\infty , {\text {hom}} } v(0) }{ \Vert v_{\infty , {\text {hom}} } \Vert ^2_2 } -1 \right) \nonumber \\&={\text {artanh}}\left( \varrho \right) - {\text {artanh}}\left( \varrho + \varTheta \right) , \end{aligned}$$

where we define

112 $$\begin{aligned} \varrho = 2 \frac{ v^T_\infty v(0) }{ \Vert v_\infty \Vert ^2_2 } -1 \end{aligned}$$

and

113 $$\begin{aligned} \varTheta = 2 \frac{ v^T_{\infty , {\text {hom}} } v(0) }{ \Vert v_{\infty , {\text {hom}} } \Vert ^2_2 } -1 - \varrho . \end{aligned}$$

The Taylor series of ${\text {artanh}}\left( \varrho + \varTheta \right)$ around $\varTheta =0$ reads

$$\begin{aligned} {\text {artanh}}\left( \varrho + \varTheta \right) = {\text {artanh}}\left( \varrho \right) + \frac{1}{1-\varrho ^2} \varTheta + {\mathcal {O}}\left( \varTheta ^2 \right) . \end{aligned}$$

Thus, we obtain from (111) that

114 $$\begin{aligned} \varUpsilon (0) - \varUpsilon _{\text {hom}}(0)&= \frac{1}{\varrho ^2-1} \varTheta + {\mathcal {O}}\left( \varTheta ^2 \right) . \end{aligned}$$

Hence, to bound the difference $\varUpsilon (0) - \varUpsilon _{\text {hom}}(0)$, we aim to bound the variable $\varTheta$. The definition of $\varTheta$ in (113) yields with (112) that

$$\begin{aligned} \varTheta&= 2 \frac{ v^T_{\infty , {\text {hom}} } v(0) }{ \Vert v_{\infty , {\text {hom}} } \Vert ^2_2 } -2 \frac{ v^T_\infty v(0) }{ \Vert v_\infty \Vert ^2_2 }\\&=2 \left( \frac{ v^T_{\infty , {\text {hom}} } }{ \Vert v_{\infty , {\text {hom}} } \Vert ^2_2 } - \frac{ v^T_\infty }{ \Vert v_\infty \Vert ^2_2 }\right) v(0). \end{aligned}$$

The Cauchy–Schwarz inequality gives that

$$\begin{aligned} \left| \varTheta \right|&\le 2 \left\Vert v(0) \right\Vert _2 \left\Vert \frac{ v_{\infty , {\text {hom}} } }{ \Vert v_{\infty , {\text {hom}} } \Vert ^2_2 } - \frac{ v_\infty }{ \Vert v_\infty \Vert ^2_2 } \right\Vert _2 \\&= 2 \frac{\left\Vert v(0) \right\Vert _2}{ \Vert v_\infty \Vert ^2_2 } \left\Vert \frac{ \Vert v_\infty \Vert ^2_2 }{ \Vert v_{\infty , {\text {hom}} } \Vert ^2_2 }v_{\infty , {\text {hom}} } - v_\infty \right\Vert _2. \end{aligned}$$

Under Assumption 2, it holds that $\Vert v(0) \Vert _2 \le \Vert v_\infty \Vert _2$, and hence

$$\begin{aligned} \left| \varTheta \right|&\le 2 \frac{1}{ \Vert v_\infty \Vert _2 } \left\Vert \frac{ \Vert v_\infty \Vert ^2_2 }{ \Vert v_{\infty , {\text {hom}} } \Vert ^2_2 }v_{\infty , {\text {hom}} } - v_\infty \right\Vert _2, \end{aligned}$$

which can be rewritten as

$$\begin{aligned} \left| \varTheta \right|&\le 2 \frac{1}{ \Vert v_\infty \Vert _2 } \left\Vert v_{\infty , {\text {hom}} } - v_\infty +\left( \frac{ \Vert v_\infty \Vert ^2_2 }{ \Vert v_{\infty , {\text {hom}} } \Vert ^2_2 } - 1 \right) v_{\infty , {\text {hom}} } \right\Vert _2. \end{aligned}$$

The triangle inequality yields that

$$\begin{aligned} \left| \varTheta \right|&\le 2\frac{\left\Vert v_{\infty , {\text {hom}} } - v_\infty \right\Vert _2}{ \Vert v_\infty \Vert _2 } + 2\frac{1}{ \Vert v_\infty \Vert _2 } \left\Vert \left( \frac{ \Vert v_\infty \Vert ^2_2 }{ \Vert v_{\infty , {\text {hom}} } \Vert ^2_2 } - 1 \right) v_{\infty , {\text {hom}} } \right\Vert _2, \end{aligned}$$

which becomes

115 $$\begin{aligned} \left| \varTheta \right|&\le 2\frac{\left\Vert v_{\infty , {\text {hom}} } - v_\infty \right\Vert _2}{ \Vert v_\infty \Vert _2 } + 2\frac{\left\Vert v_{\infty , {\text {hom}} } \right\Vert _2}{ \Vert v_\infty \Vert _2 } \left| \frac{ \Vert v_\infty \Vert ^2_2 }{ \Vert v_{\infty , {\text {hom}} } \Vert ^2_2 } -1 \right| \nonumber \\&=2\frac{\left\Vert v_{\infty , {\text {hom}} } - v_\infty \right\Vert _2}{ \Vert v_\infty \Vert _2 } + 2\frac{ \left| \Vert v_\infty \Vert ^2_2 - \Vert v_{\infty , {\text {hom}} } \Vert ^2_2 \right| }{ \Vert v_\infty \Vert _2 \left\Vert v_{\infty , {\text {hom}} } \right\Vert _2 }. \end{aligned}$$

Since, by Lemma 12, $\gamma _\text {hom}=\gamma$ and $x_{1,\text {hom}}=x_1$, Theorem 1 implies that

116 $$\begin{aligned} v_{\infty , {\text {hom}}}=\gamma x_1 + \eta _{\text {hom}} \end{aligned}$$

for some $N\times 1$ vector $\eta _{\text {hom}}$ that satisfies $\Vert \eta _{\text {hom}} \Vert _2 = {\mathcal {O}}\left( \left( R_0-1\right) ^2\right)$ as $R_0 \downarrow 1$. Thus, with (6) and (116), we obtain from (115) that

$$\begin{aligned} \left| \varTheta \right|&\le 2\frac{\left\Vert \eta - \eta _{\text {hom}} \right\Vert _2}{ \Vert v_\infty \Vert _2 } + 2\frac{ \left| 2 \gamma x_1^T \left( \eta - \eta _{\text {hom}} \right) + \Vert \eta \Vert ^2_2 - \Vert \eta _{\text {hom}} \Vert ^2_2 \right| }{ \Vert v_\infty \Vert _2 \left\Vert v_{\infty , {\text {hom}} } \right\Vert _2 }. \end{aligned}$$

Finally, since $\Vert \eta \Vert _2 = {\mathcal {O}}\left( \left( R_0-1\right) ^2\right)$, $\Vert \eta _{\text {hom}} \Vert _2 = {\mathcal {O}}\left( \left( R_0-1\right) ^2\right)$, $\gamma = {\mathcal {O}}\left( R_0-1\right)$, $\Vert v_\infty \Vert _2 = {\mathcal {O}}\left( R_0-1\right)$ and $\Vert v_{\infty , {\text {hom}} } \Vert _2 = {\mathcal {O}}\left( R_0-1\right)$, we obtain that

$$\begin{aligned} \left| \varTheta \right|&= {\mathcal {O}}\left( R_0-1 \right) \end{aligned}$$

as $R_0\downarrow 1$, which completes the proof in combination with (110) and (114). $\square$

With Lemma 13 and $\gamma = {\mathcal {O}}(R_0-1)$, we obtain from (109) that

$$\begin{aligned} \left\Vert v(t) - v_{\text {hom}}(t)\right\Vert _2&= {\mathcal {O}}\left( (R_0 - 1)^2 \right) +{\mathcal {O}}\left( (R_0 - 1)^s \right) \\&= {\mathcal {O}}\left( (R_0 - 1)^s \right) , \end{aligned}$$

since, by definition, $s = {\mathrm{min}}\{p, 2\} \le 2$. Since $\Vert v_\infty \Vert _2= {\mathcal {O}}\left( R_0 - 1 \right)$ by Theorem 1, it holds that

$$\begin{aligned} \frac{\left\Vert v(t) - v_{\text {hom}}(t)\right\Vert _2}{\Vert v_\infty \Vert _2} = {\mathcal {O}}\left( (R_0 - 1)^{s-1} \right) \end{aligned}$$

as $R_0\downarrow 1$, which completes the proof.

## Footnotes

1 In this work, we use the words node and group interchangeably.

2 The initial state vector *v*(0) is parallel to the steady-state vector $v_\infty$ if $v(0) = \alpha v_\infty$ for some scalar $\alpha \in {\mathbb {R}}$.

3 More precisely, Paré et al. (2017) assume that the adjacency matrix *A*(*t*) is time-varying but not necessarily symmetric nor binary-valued, which is equivalent to time-varying infection rates $\beta _{ij}(t)$.

4 The steady-state $v_{\infty , i}$ is the same for every node *i* in a regular graph for homogeneous spreading parameters $\beta , \delta$. Hence, the initial state $v_i(0)$ is the same for every node *i* if and only if the initial state *v*(0) is parallel to the steady-state vector $v_\infty$.

5 By convergence of the sequence of tuples $\left( B^{(n)}, S^{(n)}\right)$ to the limit $(B^*, S^*)$, we mean that, for all $\epsilon >0$, there exists an $n_0(\epsilon )\in {\mathbb {N}}$ such that both $\Vert B^{(n)} - B^* \Vert _2 < \epsilon$ and $\Vert S^{(n)} - S^* \Vert _2 < \epsilon$ holds for all $n\ge n_0(\epsilon )$.

6 Theorem 1 implies that the steady-state $v_\infty$ satisfies $\Vert v_\infty \Vert _2 = {\mathcal {O}}\left( R_0 - 1\right)$ when $R_0 \downarrow 1$. Thus, also $\Vert c(t) v_\infty \Vert _2= {\mathcal {O}}\left( R_0 - 1\right)$ at every time *t*. Thus, a *linear* convergence of the error term $\xi (t)$ to zero, i.e., $\Vert \xi (t)\Vert _2= {\mathcal {O}}\left( R_0 - 1\right)$, would not be sufficient to show that the viral state *v*(*t*) converges to $c(t)v_\infty$ when $R_0 \downarrow 1$.

7 In Prasse and Van Mieghem (2019), an analogous statement has been proved for the discrete-time version of the NIMFA equations (2).
